# How do we measure dysarthria after stroke? A systematic review to guide the core outcome set for dysarthria

**DOI:** 10.1136/bmjopen-2025-099662

**Published:** 2025-05-23

**Authors:** Claire Mitchell, Sabrina El Kouaissi, Martha Duncan-Zaleski, Audrey Bowen, Paul Conroy, Brooke-Mai Whelan, Sarah J Jane Wallace, Joshua Cheyne, Jamie J Kirkham

**Affiliations:** 1 Division of Psychology, Communication and Human Neuroscience, School of Health Sciences & Geoffrey Jefferson Brain Research Centre, The University of Manchester, Manchester, UK; 2 Manchester Centre for Health Psychology, School of Health Sciences & Geoffrey Jefferson Brain Research Centre, The University of Manchester, Manchester, UK; 3 School of Linguistic, Speech and Communication Sciences, Trinity College Dublin, Dublin, Ireland; 4 School of Health and Rehabilitation Sciences, The University of Queensland, Brisbane, Queensland, Australia; 5 Queensland Aphasia Research Centre, The University of Queensland, Brisbane, Queensland, Australia; 6 Surgical Treatment and Rehabilitation Service (STARS) Education and Research Alliance, The University of Queensland and Metro North Health, Brisbane, Queensland, Australia; 7 Library Services, University of the West of Scotland, Paisley, Renfrewshire, UK; 8 Department of Biostatistics, Manchester University, Manchester, UK

**Keywords:** Stroke, REHABILITATION MEDICINE, Systematic Review

## Abstract

**Abstract:**

**Objectives:**

A consensus study to establish a Core Outcome Set for dysarthria after stroke identified four key outcome domains that should be measured in research and clinical practice: (1) intelligibility of speech, (2) ability to participate in conversations, (3) living well with dysarthria and (4) communication partners skills and knowledge (where relevant). This review aimed to systematically identify corresponding measurement instruments and to examine their clinical utility and psychometric properties.

**Design:**

Systematic review conducted in alignment with the Preferred Reporting Items for Systematic Reviews and Meta-Analyses guidelines.

**Data sources:**

CINAHL, EMBASE, MEDLINE, PsycInfo and Cochrane Stroke Group Trials Register, CENTRAL, Linguistics and Language Behavioral Abstracts (LLBA). Major trials registers: WHO ICTRP, ISRCTN registry and ClinicalTrials.gov searched March 2024.

**Eligibility criteria for selecting studies:**

We included trials that developed or used measurement instruments for poststroke dysarthria. We identified studies that could be included in an update of the Cochrane systematic review of interventions for non-progressive dysarthria to identify what measurement instruments were used in therapy trials for poststroke dysarthria.

**Data extraction and synthesis:**

Records were screened independently by three authors. Psychometric data were extracted, by two authors, from included studies and methodological quality was evaluated using Consensus-based Standards for the selection of health Measurement Instruments (COSMIN) and Core Outcome Measures in Effectiveness Trials (COMET) guidance. Assessment of clinical utility followed Outcome Measures in Rheumatology (OMERACT) guidance.

**Results:**

Following screening, 19 publications reporting 12 measurement instruments were identified. According to COSMIN standards, all 19 publications were rated as having low, very low or unknown quality of evidence. Three measurement instruments were identified as having the most relevant clinical utility to the population, the highest quality of evidence and had the potential to measure some specific aspects from three of the four agreed domains, intelligibility, conversations and living well with dysarthria from the patient and clinician perspective. These were the Frenchay Dysarthria Assessment II, the Communication Outcomes After Stroke Scale and the Therapy Outcome Measures for Dysarthria.

**Conclusions:**

This review provides a comprehensive overview and appraisal of dysarthria measurement instruments to align with a Core Outcome Set. We only included English language-based measurement instruments. Many dysarthria measurement instruments were developed for non-stroke populations, including progressive dysarthria, with limited psychometric data for stroke. Measurement instruments with uncertain quality of evidence can still be considered for inclusion with a Core Outcome Set and three have been suggested. There is a need for further psychometric testing of these and the development of new measurement instruments to cover all aspects of intelligibility, conversations, living well with dysarthria and communication partner skills.

**PROSPERO registration number:**

CRD42022302998.

**STRENGTHS AND LIMITATIONS OF THIS STUDY**

- This systematic review used a comprehensive search for articles, including a re-run of the Cochrane review for non-progressive dysarthria, to identify measurement instruments for poststroke dysarthria.
- This review is based on Preferred Reporting Items for Systematic Reviews and Meta-Analyses guidelines.
- We used Consensus-based Standards for the selection of health Measurement Instruments (COSMIN) and Core Outcome Measures in Effectiveness Trials (COMET) guidance and the Grading of Recommendation, Assessment, Development and Evaluation approach to evaluate the strength and quality of the psychometric data as well as Outcome Measures in Rheumatology (OMERACT) guidance to evaluate clinical utility.
- Many of the measurement instruments were devised for a broader neurological population, so there is limited psychometric data for dysarthria after stroke.
- This review excluded non-English language measurement instruments and articles which may lead to language bias.

## Background

 Dysarthria, a motor speech disorder where speech is unclear or slurred, after stroke can have a devastating impact on everyday life, leading to poor psychological well-being and social isolation.^1 2^ This affects 52% of stroke survivors on admission to hospital,^3^ yet there is limited research and clinical guidance around treatment or what speech outcomes should be measured. A Core Outcome Set (COS) is the minimum set of outcomes that should be measured and reported in research. A COS for dysarthria (COS-Speech) after stroke has been agreed which identified four key outcome domains that should be measured: (1) intelligibility of speech, (2) ability to participate in conversations, (3) living well with dysarthria and (4) skills and knowledge of communication partners (where relevant).^4^ COS are more likely to be used if it is clear exactly ‘how’ these core outcomes should be measured using which measurement instruments.^5^,^7^ Therefore, the next step is to agree on how, by identifying what measurement instruments exist, their psychometric data and clinical utility and if they could measure any of the agreed core outcomes.

Measurement instruments for dysarthria after stroke do currently exist, and there are other systematic reviews of dysarthria measures in stroke. However, it is unknown whether any of these measures relate to the core outcomes identified in COS-Speech.^4 8^ This systematic review will assist in identifying all possible instruments and consider the extent to which these meet the needs of COS-Speech. Current dysarthria instruments include screening tools, diagnostic instruments and outcome measurements of speech recovery, and any of these could be included in a COS if appropriate. Screening measures are usually intended to be quick to carry out, by a health professional, and indicate whether the condition is present in a defined population.^9 10^ Diagnostic measures, carried out by a health professional, give symptoms or impairment level detail of the condition and are used to guide treatment approaches.^11^ Specifically designed outcome measures can be therapist or patient reported, offering a broader overview of recovery at activity or participation level.^12^

This study aims to systematically search for all dysarthria-related measurement instruments, examine their clinical utility and understand their psychometric data where relevant. This will help determine whether existing measurement instruments relate to the four domains identified in COS-Speech: intelligibility, conversations, living well with dysarthria and skills/knowledge of communication partners.

## Methods

The protocol for this systematic review was registered on PROSPERO in advance (ID: CRD42022302998) see online supplemental file 1 and conducted using Preferred Reporting Items for Systematic Reviews and Meta-Analyses (PRISMA) standards^13^ see checklist (online supplemental file 2). Deviations from the protocol are described here. The research team carried out two different searches for this study. The first replicated searches used in the interventions for non-progressive dysarthria Cochrane review (with permission) from May 2016 to March 2024.^14^ This was to identify new trials that would be eligible in an updated Cochrane review. Once identified, these potentially eligible studies were hand-searched for outcome measurement instruments and their related studies. In addition, the research team hand-searched the studies already included in the dysarthria Cochrane review to identify what measurement instruments had been used. Examining the dysarthria measurement instruments used in these studies identified any related publications, data or information for inclusion in this review.

In order to identify all other instruments, our second search was for existing measurement instruments for dysarthria after stroke on these electronic databases: CINAHL, EMBASE, MEDLINE, PsycInfo from earliest date to March 2024. Detailed search terms combined all possible words or phrases for: (construct) dysarthria and (population) adult stroke and (instrument) measurement (online supplemental file 3). The relevant filters for measurement properties following Consensus-based Standards for the selection of health Management Instruments (COSMIN) guidance were also used.^15^

### Inclusion/exclusion criteria

All studies of measurement instruments found in both search methods were then considered using our protocol. Population included studies of the adult population (over 18) with dysarthria due to a stroke. Aphasia-only studies and other types of brain injury causing dysarthria, including progressive neurological conditions and traumatic brain injury, were excluded.

This review included quantitative studies which examined instruments developed or used for dysarthria following stroke, including standardisation, validation, diagnostic, cohort, case-control and experimental studies, and randomised controlled trials. We excluded studies that did not examine the reliability or validity of a dysarthria measure. We were not resourced for translation, so non-English language studies or instruments in other languages were excluded. Studies where the measurement instrument could not be obtained were also excluded to reduce unnecessary analysis. In a change to the protocol, we also excluded measurement instruments that included dysarthria as part of a wider assessment of language and/or cognition and/or other physical impairments in which dysarthria-specific abilities could not be extracted, easily examined in isolation or where dysarthria was not included. If psychometric data for dysarthria only could not be extracted, this would mean data would be inaccurate, unreliable and could not be fairly compared with other studies.

### Searching

In accordance with the PRISMA checklist items 8 and 9, searching was carried out by MDZ and JC. Three reviewers (CM, HE, EL) independently assessed the titles and abstracts identified by the search using the systematic review manager Covidence (covidence.org) and managed using RAYYAN.^16^ Data were extracted by two independent reviewers (MDZ and SEK) who verified each other’s data extraction, and any queries were discussed with CM. A standardised data extraction form was developed from relevant guidance,^17 18^ to extract the following: type of measurement tool and construct measured, number and aetiology of participants on whom it was tested, test procedure and analysis method used, psychometric properties tested and the results of their analysis, clinical utility and quality assessment (COSMIN risk of bias tool^19^). Authors of all included measurement instruments were contacted to provide any unpublished data that was relevant to the reliability of validity of their instrument that could be used in this review.

Evaluation of methodological quality of the studies followed the COSMIN and Core Outcome Measures in Effectiveness Trials (COMET) guidance.^20^ We applied criteria for good measurement properties using the COSMIN taxonomy.^19 21^ We evaluated the following measurement properties for all included instruments: content validity, reliability, responsiveness, internal consistency, structural validity, measurement error, hypotheses testing, criterion validity and cross-cultural validity. The quality of the included instruments was assigned an overall rating considering the body of evidence for each instrument, which includes the number of studies, the methodological quality of the studies and the consistency of the results^22^ using the Grading of Recommendations Assessment Development and Evaluation (GRADE).^19 23^ The ratings were classified as follows: high quality (consistent findings in multiple studies of at least good quality OR one study of excellent quality AND a total sample size of ≥100 patients); moderate (conflicting findings in multiple studies of at least good quality OR consistent findings in multiple studies of at least fair quality OR one study of good quality AND a total sample size of ≥50 patients); low (conflicting findings in multiple studies of at least fair quality OR one study of fair quality AND a total sample size of ≥30 patients); very low (only studies of poor quality OR a total sample size of <30 patients); unknown if no studies can be found.

Evaluation of clinical utility was carried out following guidance from Outcome Measures in Rheumatology (OMERACT),^24^ considering these relevant issues: training required to deliver and/or interpret; number of people needed to carry out; completion time; accessible for people with aphasia and commercial availability and cost.

### Patient and public involvement

The planned systematic review was discussed with our patient and public involvement group of stroke survivors living with dysarthria, Healing, Empowering and Recovering from Dysarthria. We informed them of the review process and discussed the process and results with them. The group suggested we should highlight the small number of stroke survivors involved in developing measurement instruments. They also thought it was important to raise the lack of measurement instruments to examine psychological well-being when living with dysarthria. They did not interpret the results or contribute to the writing or editing of this document.

## Results

The Cochrane search identified 8286 studies after duplicates removed. These were examined as to whether they met the criteria for a Cochrane review. Twelve full-text papers were considered, with 10 meeting the Cochrane inclusion criteria and relevant measurement instruments used in these 10 studies were extracted. Eighteen studies related to these instruments were then considered for inclusion in this review of measures.

**Figure 1:**
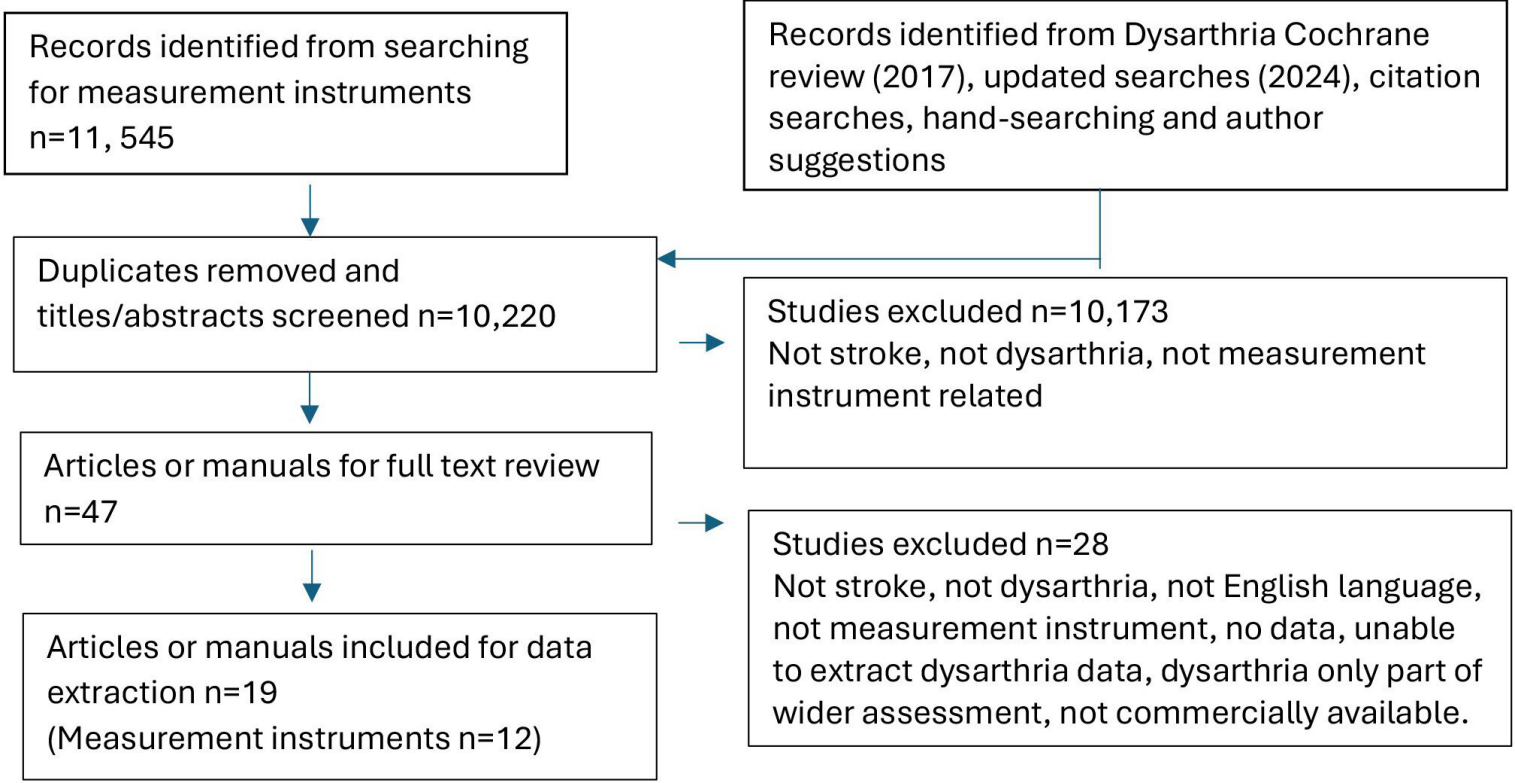
Preferred Reporting Items for Systematic Reviews and Meta-Analyses flow chart to summarise the screening process.

The second search for all dysarthria measurement instruments found a total of 10 220 after duplicates removed. The 18 potential Cochrane studies were included for screening. Following systematic title and abstract screening, 47 full text papers were considered (see figure 1, PRISMA flow chart). We excluded 28 articles related to 11 measurement instruments (online supplemental file 4) due to a variety of reasons: not English language, not tested on poststroke dysarthria, not designed to examine dysarthria, not available, not a measurement instrument, no psychometric data at all.

We included 19 papers and manuals; these provided details on 12 measurement instruments for dysarthria after stroke and involved 137 stroke survivors with dysarthria. Of these 12 measures, one is a screening instrument,^25^ four are diagnostic clinician-rated instruments^26^,^29^ and seven were outcome measurement instruments with five of these patient reported^30^,^35^ and two clinician reported.^12 36 37^ Of the 12 included measurement instruments, only one was specifically developed for stroke survivors; 11 were developed more broadly for people with other neurological dysarthria. The psychometric data available for the included measurement instruments and the related studies are detailed in online supplemental file 5.

### Measurement instruments designed to screen for dysarthria

We included one combined screening instrument to identify the presence of dysarthria (Maximum Phonation Time (MPT) and Maximum Repetition Rate (MRR)). We found one other screening instrument, the National Institute for Health Stroke Scale (NIHSS), which we excluded from this review (online supplemental file 4) as this instrument is designed to rate neurological function with signs and symptoms of stroke, with dysarthria being one element of this.^38^ Dysarthria data could not be extracted from the many NIHSS studies examining validity and reliability data and were excluded on that basis.

MPT involves asking someone to hold a vowel for as long as possible and is used alongside MRR, where someone repeats syllables as quickly as possible. The one study examining MPT and MRR included 26 stroke survivors with dysarthria, and the psychometric data suggest this is an unreliable measure of identifying dysarthria with low sensitivity and specificity.^25^ The quality of evidence is very low due to the small numbers, and the data suggest that both MPT and MMR are not valid or reliable measures of the presence of dysarthria from the data.

### Measurement instruments designed for detailed diagnostic assessment of dysarthria

We identified four diagnostic measurement instruments for dysarthria. (1) The Assessment of Intelligibility of Dysarthric Speech (AIDS) is a measure of word and sentence reading intelligibility as rated by two speech and language therapists resulting in a numerical score of intelligibility.^39^ (2) The Frenchay Dysarthria Assessment V. 2 (FDA II) examines oro-facial and other speech-related musculature. Movement, including reading words, sentences and conversation using a rating scale compared with ‘healthy’ movement.^27^ (3) The Iowa Oral Performance Instrument (IOPI) is a measure of tongue and lip strength using a pressure reading monitor using a small plastic bulb.^28^ (4) The ‘Reading Passages’ assessment is designed for an individual to read a passage out loud which can then be analysed for any difficulties with intonation, sounds and complex words.^29^

These four assessments have been developed on a small number of 25 stroke survivors with dysarthria (AIDS n=9, FDA n=4, IOPI n=3, Reading Passages n=9). The FDA, AIDS and IOPI provide psychometric data (online supplemental file 5). The AIDS and the FDA both report reliable interjudge and intrajudge ratings but on extremely small data sets. The IOPI-reported test-retest scores only with no evaluation of the validity or reliability of the IOPI for measuring dysarthria after stroke. The ‘Reading Passages’ research did not give data relating to the reliability and validity of assessing dysarthria.

### Measurement instruments designed for outcome measurement of dysarthria

We identified seven measures intended to examine outcomes for stroke survivors with dysarthria; (1) the Communication Outcomes After Stroke Scale (COAST),^35^ (2) the Communicative Participation Item Bank (CPIB),^31 40 41^ (3) the Dysarthria Impact Profile (DIP),^32^ (4) the Questionnaire on Acquired Speech Disorders (QASD),^33^ (5) the Quality of Life in a Dysarthric Speaker (QOL-DyS),^34^ (6) the O’Halloran, Hickson, Worrall (OHW) Speech, Language and Cognitive Communicative Scales ^42^ and (7) Therapy Outcome Measures for Dysarthria (D-TOMs).^12^ Five of these, the COAST, CPIB, DIP, QASD and QOL-DyS, were designed as patient-reported outcome measures and two, OHW and D-TOMs, were therapist-reported measures.

Two of the measures (COAST and Dysarthria TOMs) were evaluated on the same study population of 102 stroke survivors with aphasia and/or dysarthria, with 30 diagnosed as having dysarthria. The COAST was found to have good internal consistency and test-retest reliability but was not repeated over time, so there is no measure of responsiveness. The D-TOMs were tested on the same population as the COAST as part of a bigger study ACTNoW,^30^ which included the same 102 participants (30 with dysarthria). The TOMs aphasia/dysarthria was found to have high intra-rater and inter-rater agreement with high levels of conversation reliability. The TOMs were shown to have well above commonly accepted levels for reliability data. The COAST and the TOMs would be considered to have low levels of quality of evidence. The total number of people examined was >100 but only 30 of these had dysarthria.

The CPIB, QASD, QOL-DyS, DIP and OHW were only tested in stroke populations of n=18, n=1, n=7, n=7 and n=11, respectively, due to these being designed for a wider adult neurological population. They could only be rated as a very low quality of evidence due to the review’s focus on stroke-specific data.

### Clinical utility

All diagnostic assessments were designed to be carried out by a qualified speech and language therapist or speech specialist professional.

MPT/MRR scores, although quick to carry out, cannot be considered to have clinical utility as there are no comparable ‘healthy’ or ‘typical’ MPT or MRR scores. The AIDS has limited clinical utility due to the need for two people to rate the 220 words needing to be transcribed and judged by another rater, which makes this resource heavy, reducing clinical utility. The FDA is carried out by one person and uses clear descriptors to rate specific speech musculature and intelligibility. No specified time is given in the manual. IOPI and ‘Reading Passages’ can both be carried out by one person. No time is given for either carrying out or interpreting the data. The COAST did give an estimate of time taken to complete of 20/25 min (median time).^35^ The D-TOMs also gave an estimated time of just a few minutes. The DIP does not give a time to complete, and the OHW has to be carried out in conjunction with the Inpatient Functional Communication Interview (IFCI), so it is difficult to predict time to complete. The QASD and QOL-DyS do not specify completion times, but authors report that this was not an onerously long task.

In terms of clinical utility in a population where dysarthria and aphasia co-occur, all assessments were considered for aphasia accessibility. The MPT/MRR could potentially be used for people with aphasia. The AIDS and the ‘Reading Passages’ approach relies on reading words and sentences aloud, so it would not be suitable for people with aphasia. Most of the FDA is aphasia accessible, but there is a small section related to intelligibility that involves reading words and sentences. This is not essential for rating intelligibility in the FDA. The IOPI is not language dependent, so it would be suitable. The COAST would be suitable for people with both aphasia and dysarthria and is a visual analogue scale with pictures. The other four, CIPB, DIP, QASD, QOL-DyS, involve written sentences, so are not aphasia accessible, although the CIPB can be suitable for people with aphasia with support. The OHW and D-TOMs are therapist-reported measures of outcome, so accessible to all.

All of the instruments included were commercially available, and any not commercially available had already been excluded. Costs for the AIDs and FDA are under £200, but the IOPI is a greater outlay of £1500 and requires purchase of single-use plastic bulbs to measure tongue and lip movement. All others were free.

### Summary

In summary, when considering the clinical utility, psychometric data and purpose of the measure, we identified the following three measures as having potential to be included with the COS for dysarthria (table 1): the FDA II, the COAST and the D-TOMs. These three measurement instruments were found to have the best clinical utility for people with dysarthria after stroke, with no or limited training to use, only one tester and were commercially available for use, with reasonable or no costs. They all had at least some psychometric data for the poststroke dysarthric population, and although the quality of evidence was low or very low, the clinical utility supported consideration for COS-Speech.

**Table 1:** Evidence for and clinical utility of measurement instruments for dysarthria

Measurement instrument	Training to deliver or interpret?	Only one person needed	Aphasia accessible	Commercially available or easily accessible	Cost	Quality of evidence	Consider for COS-Speech?
Screening instrument							
MPT & MRR	Yes	Yes	Yes	Yes	None	Very low	No
Diagnostic measurement instrument							
AIDS	Yes	No	No	Yes	£155	Very low	No
FDA	Yes	Yes	Partly	Yes	£199	Very low	Yes
IOPI	Yes	Yes	Yes	Yes	£1500	Unknown	No
Reading passages	Yes	Yes	No	Yes	None	Unknown	No
Outcome measurement instrument patient reported							
COAST	No	Yes	Yes	Yes	None	Low	Yes
CPIB	No	Yes	Partly	Yes	None	Very low	No
DIP	No	Yes	No	Yes	None	Very low	No
QASD	No	Yes	No	Yes	None	Very low	No
QOL-DyS	No	Yes	No	Yes	None	Very low	No
Outcome measurement instrument therapist reported							
OHW	No	Yes	Yes	Yes	None	Very low	No
TOMs	No	Yes	Yes	Yes	None	Low	Yes

AIDS, Assessment of Intelligibility of Dysarthric Speech; COAST, Communication Outcomes After Stroke Scale; COS, Core Outcome Set; CPIB, Communicative Participation Item Bank; DIP, Dysarthria Impact Profile; FDA, Frenchay Dysarthria Assessment; IOPI, Iowa Oral Performance Instrument; MPT, Maximum Phonation Time; MRR, Maximum Repetition Rate; OHW, O’Halloran, Hickson, Worrall; QASD, Questionnaire on Acquired Speech Disorder; QOL-DyS, Quality of Life ina Dysarthric Speaker; TOMs, Therapy Outcome Measures.

## Discussion

This is the first systematic review of outcome measurement instruments for dysarthria after stroke to align with the newly defined COS-Speech domains.^4^ The systematic review included 12 possible measurement instruments for consideration in the COS, of which we recommend three instruments: FDA, COAST and D-TOMs. COS-speech identified four outcome domains (ie, what should be measured) of intelligibility, conversations, living well with dysarthria and communication partners which guided our search for instruments (ie, how to measure them). The FDA includes some elements of intelligibility and intelligibility in conversation to a limited extent as rated by the therapist, but broader measures of intelligibility in different contexts still need to be developed. The COAST also considers some elements of intelligibility, being able to take part in conversations and living well with dysarthria from the patient perspective, but does not cover some of the nuanced issues around well-being that were highlighted in COS-Speech. The D-TOMs broadly cover intelligibility and some elements of living well with dysarthria as rated by the therapist, but as with all the suggested measures, would benefit from further validating in the dysarthric poststroke population. All 12 instruments identified are described in this paper in terms of quality of data, psychometric data and clinical utility, providing a rationale for our recommendations of three. The included measures were mostly designed for all types of neurological dysarthria, not poststroke dysarthria, which had an impact on what data could be included in and meant that many measures were rated as low or very low quality. This is not necessarily a reflection on the quality of these measures more broadly in the adult neurological population, and we would like to make this clear: our intention is specifically examining dysarthria outcomes after stroke.

Screening instruments are designed to identify quickly and easily whether a set of symptoms is present, in this case dysarthria, in a particular population, which for this study was stroke.^10^ We excluded the widely used NIHSS from the review, due to being unable to extract specific dysarthria data. Another frequently used screening test for dysarthria, phonation and sound repetition (MPT and MRR), was included in the review and data extracted but was excluded from consideration in the COS due to the very low quality of evidence and highly unreliable psychometric data.^25^ Ziegler^25^ suggests there is no evidence that these tests have any use in identifying dysarthria, and they have no place in clinical practice for their ongoing use, but there can be challenges around stopping traditionally used methods.^43^

Diagnostic instruments are intended to provide an in-depth comprehensive overview of someone’s speech difficulties across multiple domains to guide treatment. Developing diagnostic speech instruments is hugely challenging, and the work to establish reliability and validity, as well as developing the assessment itself, means it is not unusual to have limited psychometric data. The measurement instruments we identified had been designed for the whole dysarthria population, which can include all adult acquired and congenital neurological conditions, progressive and non-progressive. One of the most commonly used clinical dysarthria assessments in the UK is the FDA.^44^ This measurement instrument, which looks at impairment and activity level difficulties, has been developed and tested on a much wider population than just stroke, which may be of higher quality data than we could consider in this review examining stroke-only data. This instrument has clinical utility and may be considered for inclusion with the COS for speech and warrants further discussion. All other measures could not be considered for the COS due to having very low quality of evidence with limited clinical utility for aphasia.

Outcome measurement instruments have a different purpose from screening and diagnostic testing, designed to examine the broader perspective of how the individual is functioning in everyday life and quality of life.^45^ In this review, we have included instruments that were designed specifically to look at outcomes. We concluded that the CPIB, DIP, QASD and QOL-DyS were all designed for a broader neurological dysarthric population rather than stroke. These could not be considered in their current format due to limited testing in the stroke population. We also ruled out the OHW, which was reliant on another measure (IFCI) and had some outstanding concerns regarding how reliable and valid it was.^36^ The COAST and TOMs could both be considered of potential use with the COS-Speech. These two measures had low quality of evidence but were both aphasia accessible, with good clinical utility. The COMET and COSMIN guidance does suggest that content validity is a key factor in judging whether the instrument should be considered for a COS.^20^ By applying the guidance too stringently, we would be left with no potential measurement instruments for COS-Speech, and pragmatic decisions may be necessary.

From this systematic review, we have three measurement instruments that could be considered for inclusion with COS-Speech: the FDA, the COAST and the D-TOMs. The inclusion criterion that specified poststroke dysarthria only may be a limitation, as well as a strength of this review. Regarding the former, this may have excluded some assessments that could be of use with this population and would benefit from further stroke-specific psychometric data such as the CPIB and CETI-M. There may be clinically useful measures for dysarthria after stroke in other languages that have also been excluded from this study. We continue to follow the example set by the COS for people with aphasia after stroke and will learn from their progress.^46^ The three identified measures are all easily accessible and match some aspects of the outcome domains identified. They are already commonly used in clinical practice, and we encourage this ongoing use. We are currently using these as part of a longitudinal research study to explore the trajectory of speech recovery after stroke and will report on participants’ view of acceptability and accuracy. For future research and clinical practice, we need to develop measurement instruments to further examine intelligibility and conversation in everyday settings, considering the different requirements in these contexts and providing a more detailed focus on activity and participation level that goes beyond the FDA. We need to examine what elements of living well with dysarthria are adequately covered by the COAST and what might be missing, as this might be possible to adapt. We also need to develop or adapt any measurement instruments for communication partners to understand if they would be applicable for people with dysarthria after stroke and may be able to adapt measures being developed for the aphasia population.

## Supplementary material

10.1136/bmjopen-2025-099662 online supplemental file 1

10.1136/bmjopen-2025-099662 online supplemental file 2

10.1136/bmjopen-2025-099662 online supplemental file 3

10.1136/bmjopen-2025-099662 online supplemental file 4

10.1136/bmjopen-2025-099662 online supplemental file 5

## Data Availability

Data are available upon reasonable request.
