# Supplementary material for: How do we measure dysarthria after stroke? A systematic review to guide the core outcome set for dysarthria
Source: BMJ Open. 2025 May 23;15(5):e099662. doi: 10.1136/bmjopen-2025-099662 (PMC12104885; doi:10.1136/bmjopen-2025-099662)
Supplement: online supplemental file 1 [file bmjopen-15-5-s001.pdf]

A systematic review of the psychometric properties and clinical utility of instruments measuring dysarthria after stroke (PROSPERO International prospective register of systematic reviews)

Review question

What instruments/scales/tests/questionnaires are there to measure speech recovery for people with dysarthria after stroke?

What are the psychometric properties (reliability, validity, sensitivity to change) of these instruments?

What is the clinical utility (time taken to administer, cost, specialist training, portability of measure) of these measures?

Searches

PRISMA guided systematic review. The Cochrane review Interventions for dysarthria due to stroke and other adult-acquired, non-progressive brain injury will be searched for instruments of any sort to measure speech recovery (this recent update includes searches of The Cochrane Library so these will not be searched again). Electronic databases (CINAHL, EMBASE, MEDLINE, PsycInfo, Pubmed) will be searched from their earliest date to February 2022.

The search terms will include terms related to assessment or measurement or outcome measure or measurement tool or scale or questionnaire or profile or quality. The search strategy will follow the guidance for database specific search filters to find measurement properties of instruments developed by Terwee (1) as described on the COSMIN website

Types of study to be included

Any studies that show the development of a measure for dysarthria after stroke.

Condition or domain being studied

Dysarthria after stroke is a motor speech difficulty that means speech can sound different in many ways. The severity ranges from minimal changes to sounds that cannot be understood. Speech can sound slurred or unclear, speech can be slow, it can affect the quality of voice, volume, nasality and tone. The focus is on what instruments are used to measure how stroke survivors are affected by dysarthria.

Participants/population

Adults over the age of 18 who are stroke survivors who have dysarthria of any severity and with or without co-occurring aphasia. No restrictions on time post stroke, severity of dysarthria or stroke.

Intervention/exposure

Any instrument/measure/test/scale/questionnaire for dysarthria after stroke. This will include patient-reported outcomes, clinician-reported outcomes and performance-based outcome measures.

Comparator/control

Not applicable

Types of study to be included

All studies of the instruments used for or developed for measuring dysarthria after stroke to be included. Adult stroke only. English language studies only.

Excluded – studies for dysarthria of progressive conditions.

- Studies where there was no information about how the device could be obtained or was clearly not commercially available.

Primary Outcome

To determine what instruments exist for measuring dysarthria after stroke.

Secondary Outcomes

To determine the psychometric properties of these instruments used for dysarthria after stroke.

To determine the clinical utility of these instruments used for dysarthria after stroke.

Data extraction

The titles and abstracts of the references retrieved from our searches will be screened to identify the relevant records. Potentially eligible full-text references will be retrieved and assessed for inclusion/exclusion against the criteria we set out.

A standardised form will be used for extraction of the data from the included studies. Two reviewers CM and MDZ will carry out the selection and extraction process independently and any disagreements will be discussed between them to gain agreement.

Risk of bias (quality) assessment

Risk of bias will be carried out following the COSMIN Risk of Bias tool (2) as well as considering practicable useability (based on time taken to administer, cost, specialist training, portability of measure).

Strategy for data synthesis

The results of all studies on each measurement property for each measure will be summarised qualitatively and the overall quality of the evidence will be determined using a modified GRADE approach based on the risk of bias and clinical useability.

Recommendations will consider the quality of findings and quality of the evidence.

Analysis of subgroups or subsets

Not appropriate to specify subgroups.

General information

Systematic review

Rehabilitation

Language English

Country UK

Key words: systematic review, psychometric properties, dysarthria, stroke

Anticipated start date

February 2022

Completion date

February 2023

References:

1. Terwee CB, Jansma EP, Riphagen II, de Vet HCW. Development of a methodological PubMed search filter for finding studies on measurement properties of measurement instruments. Quality of Life Research. 2009;18(8):1115-23.
2. Mokkink LB, Terwee CB, Patrick DL, Alonso J, Stratford PW, Knol DL, et al. The COSMIN checklist for assessing the methodological quality of studies on measurement properties of health status measurement instruments: an international Delphi study. Quality of Life Research. 2010;19(4):539-49.
3. Mokkink LB, Terwee CB, Gibbons E, Stratford PW, Alonso J, Patrick DL, et al. Inter-rater agreement and reliability of the COSMIN (COnsensus-based Standards for the selection of health status Measurement Instruments) checklist. BMC Medical Research Methodology. 2010;10(1):82.
