# Supplementary material for: How do we measure dysarthria after stroke? A systematic review to guide the core outcome set for dysarthria
Source: BMJ Open. 2025 May 23;15(5):e099662. doi: 10.1136/bmjopen-2025-099662 (PMC12104885; doi:10.1136/bmjopen-2025-099662)
Supplement: online supplemental file 3 [file bmjopen-15-5-s003.pdf]

Supplementary file 3: Search strategies and filters

Search strategies

The MEDLINE Ovid database search strategy was developed by an experienced Information Specialist (JDC) to identify studies for analysis. It contained 57 separate lines and utilised a combination of Medical Subject Headings (MeSH) and uncontrolled vocabulary/keywords to identify relevant literature. The search criteria encompassed terms related to adult stroke survivors (lines 1-9) and measurement instruments for post-stroke dysarthria (lines 11-22 and 23-56 respectively).

The search strategy included The Cochrane Stroke Group's approach for identifying 'stroke' studies in respective databases and other resources[1, 2]. The 'stroke' terms are adapted from Cheyne (2023) for this review. The search filter used for identifying studies on measurement properties in MEDLINE Ovid was adapted from a validated search filter (lines 23-56) developed by Terwee et al. for PubMed[3]. This filter was appended on the search strategy designed for this review, building upon an existing dysarthria MEDLINE Ovid strategy that was updated in October 2022.

We searched the following databases:

- MEDLINE Ovid (from 1946) (Appendix 1)
- Embase Ovid (from 1974)
- CINAHL EBSCO (Cumulative Index to Nursing and Allied Health Literature; from 1937)
- APA PsycINFO Ovid (from 1806)

MEDLINE Ovid Search Strategy

1. cerebrovascular disorders/ or exp basal ganglia cerebrovascular disease/ or brain ischemia/ or brain infarction/ or brain stem infarctions/ or cerebral infarction/ or infarction, anterior cerebral artery/ or infarction, middle cerebral artery/ or infarction, posterior cerebral artery/ or hypoxia-ischemia, brain/ or ischemic attack, transient/ or vertebrobasilar insufficiency/ or carotid artery diseases/ or exp carotid artery injuries/ or carotid artery thrombosis/ or carotid stenosis/ or cerebral small vessel diseases/ or intracranial arterial diseases/ or cerebral arterial diseases/ or intracranial aneurysm/ or intracranial arteriosclerosis/ or exp intracranial arteriovenous malformations/ or exp "intracranial embolism and thrombosis"/ or intracranial hemorrhages/ or cerebral hemorrhage/ or cerebral intraventricular hemorrhage/ or subarachnoid hemorrhage/ or stroke/ or hemorrhagic stroke/ or exp ischemic stroke/ or vasospasm, intracranial/ or brain injuries/ or brain injury, chronic/

2. stroke rehabilitation/

3. (stroke or poststroke or post-stroke or cerebrovasc$ or (cerebr$ adj3 vasc$) or CVA$ or apoplectic or apoplex$ or (transient adj3 isch?emic adj3 attack) or tia$ or SAH or AVM or ESUS or ICH or (cerebral small vessel adj3 disease$)).tw.

4. ((cerebr$ or cerebell$ or arteriovenous or vertebrobasil$ or interhemispheric or hemispher$ or intracran$ or intracerebral or infratentorial or supratentorial or MCA$ or ((anterior or posterior) adj3 circulat$) or lenticulostriate or ((basilar or brachial or vertebr$) adj3 arter$)) adj3 ((blood adj5 clot$) or disease$ or damage$ or disorder$ or disturbance or dissection or lesion or syndrome or arrest or accident or lesion or vasculopathy or insult or attack or injury or insufficiency or malformation or obstruct$ or anomal$)).tw.

5. ((cerebr$ or cerebell$ or arteriovenous or vertebrobasil$ or interhemispheric or hemispher$ or intracran$ or corpus callosum or intracerebral or intracortical or intraventricular or periventricular or posterior fossa or infratentorial or supratentorial or MCA$ or ((anterior or posterior) adj3 circulation) or basal ganglia or ((basilar or brachial or vertebr$) adj3 arter$) or space-occupying or brain ventricle$ or lacunar or cortical or ocular) adj3 (isch?emi$ or infarct$ or thrombo$ or emboli$ or occlus$ or hypoxi$ or vasospasm or obstruct$ or vasoconstrict$)).tw.

6. ((cerebr$ or cerebell$ or vertebrobasil$ or interhemispheric or hemispher$ or intracran$ or corpus callosum or intracerebral or intracortical or intraventricular or periventricular or posterior fossa or infratentorial or supratentorial or MCA$ or ((anterior or posterior) adj3 circulation) or basal ganglia or ((basilar or brachial or vertebr$) adj3 arter$) or space-occupying or brain ventricle$ or subarachnoid$ or arachnoid$) adj3 (h?emorrhag$ or h?ematom$ or bleed$)).tw.

7. ((carotid or cerebr$ or cerebell$ or intracranial or ((basilar or brachial or vertebr$) adj3 arter$)) adj3 (aneurysm or malformation$ or block$ or dysplasia or disease$ or bruit or injur$ or narrow$ or obstruct$ or occlusion or constriction or presclerosis or scleros$ or stenos$ or atherosclero$ or arteriosclero$ or plaque$ or thrombo$ or embol$ or arteriopathy)).tw.

8. hemiplegia/ or paresis/ or exp gait disorders, neurologic/

9. (hemipleg$ or hemipar$ or paresis or paretic).tw.

10. or/1-9

11. speech disorders/ or articulation disorders/ or dysarthria/ or voice disorders/ or aphonia/ or dysphonia/ or communication disorders/

12. speech articulation tests/

13. (dysarth$ or dysphon$ or anarth$ or dyspros$ or aphon$ or dysfluen$ or stutter$ or stammer$).tw.

14. ((speech or articul$ or disarticul$ or phonat$ or phonolog$ or voice or vocal or prosod$ or intonat$ or respirat$ or communicat$ or fluen$) adj5 (disorder$ or impair$ or problem$ or difficult$)).tw.

15. (speech adj5 (slow$ or weak$ or imprecis$ or intelligibil$ or unintelligibil$ or accuracy or fatigue)).tw.

16. mouth/ or larynx/ or laryngeal muscles/ or pharynx/ or pharyngeal muscles/ or facial muscles/ or palatal muscles/

17. (mouth or tongue or lingual or palat$ or laryn$ or pharyn$ or orofacial or oro-facial or face musc$ or facial musc$).tw.

18. 16 or 17

19. movement disorders/ or ataxia/ or dystonia/ or dystonic disorders/ or hyperkinesis/ or hypokinesia/ or muscle hypertonia/ or muscle hypotonia/ or muscle weakness/ or muscular diseases/ or muscle spasticity/

20. (atax$ or dyston$ or hyperkin$ or hypokin$ or hypoton$ or hyperton$ or flaccid$ or spastic$).tw.

21. 19 or 20

22. 11 or 12 or 13 or 14 or 15 or (21 and 18)

23. (instrumentation or methods).fs.

24. (Validation Studies or Comparative Study).pt.

25. exp Psychometrics/

26. psychometr*.ti,ab.

27. (clinimetr* or clinometr*).tw.

28. exp "Outcome Assessment (Health Care)"/

29. outcome assessment.ti,ab.

30. outcome measure*.tw.

31. exp Observer Variation/

32. observer variation.ti,ab.

33. exp Health Status Indicators/

34. exp "Reproducibility of Results"/

35. reproducib*.ti,ab.

36. exp Discriminant Analysis/

37. (reliab* or unreliab* or valid* or coefficient or homogeneity or homogeneous or "internal consistency").ti,ab.

38. (cronbach* and (alpha or alphas)).ti,ab.

39. (item and (correlation* or selection* or reduction*)).ti,ab.

40. (agreement or precision or imprecision or "precise values" or test-retest).ti,ab.

41. (test and retest).ti,ab.

42. (reliab* and (test or retest)).ti,ab.

43. (stability or interrater or inter-rater or intrarater or intra-rater or intertester or inter-tester or intratester or intra-tester or interobserver or inter-observer or intraobserver or intraobserver or intertechnician or inter-technician or intratechnician or intra-technician or interexaminer or inter-examiner or intraexaminer or intra-examiner or interassay or interassay or intraassay or intra-assay or interindividual or inter-individual or intraindividual or intra-individual or interparticipant or inter-participant or intraparticipant or intra-participant or kappa or kappa's or kappas or repeatab*).ti,ab.

44. ((replicab* or repeated) and (measure or measures or findings or result or results or test or tests)).ti,ab.

45. (generaliza* or generalisa* or concordance).ti,ab.

46. (intraclass and correlation*).ti,ab.

47. (discriminative or "known group" or factor analysis or factor analyses or dimension* or subscale*).ti,ab.

48. (multitrait and scaling and (analysis or analyses)).ti,ab.

49. (item discriminant or interscale correlation* or error or errors or "individual variability").ti,ab.

50. (variability and (analysis or values)).ti,ab.

51. (uncertainty and (measurement or measuring)).ti,ab.

52. ("standard error of measurement" or sensitiv* or responsive*).ti,ab.

53. ((minimal or minimally or clinical or clinically) and (important or significant or detectable) and (change or difference)).ti,ab.

54. (small* and (real or detectable) and (change or difference)).ti,ab.

55. (meaningful change or "ceiling effect" or "floor effect" or "Item response model" or IRT or Rasch or "Differential item functioning" or DIF or "computer adaptive testing" or "item bank" or "cross-cultural equivalence").ti,ab.

56. or/23-55

57. 10 and 22 and 56

Embase Ovid Search strategy

1. cerebrovascular disorders/ or cerebral hemorrhage/ or exp cerebral ischemia/ or cerebral small vessel disease/ or cerebrovascular accidents/ or subarachnoid hemorrhage/

2. (stroke or poststroke or post-stroke or cerebrovasc$ or (cerebr$ adj3 vasc$) or CVA$ or apoplectic or apoplex$ or (transient adj3 isch?emic adj3 attack) or tia$ or SAH or AVM or ESUS or ICH or (cerebral small vessel adj3 disease$)).tw.

3. ((cerebr$ or cerebell$ or arteriovenous or vertebrobasil$ or interhemispheric or hemispher$ or intracran$ or intracerebral or infratentorial or supratentorial or MCA$ or ((anterior or posterior) adj3 circulat$) or lenticulostriate or ((basilar or brachial or vertebr$) adj3 arter$)) adj3 ((blood adj5 clot$) or disease$ or damage$ or disorder$ or disturbance or dissection or lesion or syndrome or arrest or accident or lesion or vasculopathy or insult or attack or injury or insufficiency or malformation or obstruct$ or anomal$)).tw.

4. ((cerebr$ or cerebell$ or arteriovenous or vertebrobasil$ or interhemispheric or hemispher$ or intracran$ or corpus callosum or intracerebral or intracortical or intraventricular or periventricular or posterior fossa or infratentorial or supratentorial or MCA$ or ((anterior or posterior) adj3 circulation) or basal ganglia or ((basilar or brachial or vertebr$) adj3 arter$) or space-occupying or brain ventricle$ or lacunar or cortical or ocular) adj3 (isch?emi$ or infarct$ or thrombo$ or emboli$ or occlus$ or hypoxi$ or vasospasm or obstruct$ or vasoconstrict$)).tw.

5. ((cerebr$ or cerebell$ or vertebrobasil$ or interhemispheric or hemispher$ or intracran$ or corpus callosum or intracerebral or intracortical or intraventricular or periventricular or posterior fossa or infratentorial or supratentorial or MCA$ or ((anterior or posterior) adj3 circulation) or basal ganglia or ((basilar or brachial or vertebr$) adj3 arter$) or space-occupying or brain ventricle$ or subarachnoid$ or arachnoid$) adj3 (h?emorrhag$ or h?ematom$ or bleed$)).tw.

6. hemiparesis/ or hemiplegia/

7. (hemipleg$ or hemipar$ or paresis or paretic).tw.

8. or/1-7

9. dysarthria/ or articulation disorders/

10. dysphonia/ or speech disorders/

11. (dysarth$ or dyphon$ or anarth$ or dyspros$ or aphon$ or dysfluen$ or stutter$ or stammer$).tw.

12. ((speech or articul$ or disarticul$ or phonat$ or phonolog$ or voice or vocal or prosod$ or intonat$ or respirat$ or communicat$ or fluen$) adj5 (disorder$ or impair$ or problem$ or difficult$)).tw.

13. (speech adj5 (slow$ or weak$ or imprecis$ or intelligibil$ or unintelligibil$ or accuracy or fatigue)).tw.

14. "mouth (anatomy)"/ or tongue/

15. respiratory system/ or exp larynx/ or pharynx/

16. vocal cords/

17. facial muscles/

18. (mouth or tongue or lingual or palat$ or laryn$ or pharyn$ or orofacial or oro-facial or face musc$ or facial musc$).tw.

19. 14 or 15 or 16 or 17 or 18

20. muscular disorders/ or exp movement disorders/ or ataxia/ or bradykinesia/ or dyskinesia/ or hyperkinesis/ or neuromuscular disorders/ or spasms/ or muscle spasms/

21. (atax* or dyston* or hyperkin* or hypokin* or hypoton* or hyperton* or flaccid* or spastic*).tw.

22. 20 or 21

23. 19 and 22

24. 9 or 10 or 11 or 12 or 13 or 23

25. exp psychometrics/

26. statistics/

27. methodology/ or causal analysis/ or cohort analysis/ or content analysis/ or data collection/ or empirical methods/

28. exp experimental design/

29. psychometr*.ti,ab.

30. (clinimetr* or clinometr*).tw.

31. outcome assessment.ti,ab.

32. outcome measure*.tw.

33. observer variation.ti,ab.

34. reproducib*.ti,ab.

35. (reliab* or unreliab* or valid* or coefficient or homogeneity or homogeneous or "internal consistency").ti,ab.

36. (cronbach* and (alpha or alphas)).ti,ab.

37. (item and (correlation* or selection* or reduction*)).ti,ab.

38. (agreement or precision or imprecision or "precise values" or test-retest).ti,ab.

39. (test and retest).ti,ab.

40. (reliab* and (test or retest)).ti,ab.

41. (stability or interrater or inter-rater or intrarater or intra-rater or intertester or inter-tester or intratester or intra-tester or interobserver or inter-observer or intraobserver or intraobserver or intertechnician or inter-technician or intratechnician or intra-technician or interexaminer or inter-examiner or intraexaminer or intra-examiner or interassay or interassay or intraassay or intra-assay or interindividual or inter-individual or intraindividual or intra-individual or

interparticipant or inter-participant or intraparticipant or intra-participant or kappa or kappa's or kappas or repeatab*).ti,ab.

42. ((replicab* or repeated) and (measure or measures or findings or result or results or test or tests)).ti,ab.

43. (generaliza* or generalisa* or concordance).ti,ab.

44. (intraclass and correlation*).ti,ab.

45. (discriminative or "known group" or factor analysis or factor analyses or dimension* or subscale*).ti,ab.

46. (multitrait and scaling and (analysis or analyses)).ti,ab.

47. (item discriminant or interscale correlation* or error or errors or "individual variability").ti,ab.

48. (variability and (analysis or values)).ti,ab.

49. (uncertainty and (measurement or measuring)).ti,ab.

50. ("standard error of measurement" or sensitiv* or responsive*).ti,ab.

51. ((minimal or minimally or clinical or clinically) and (important or significant or detectable) and (change or difference)).ti,ab.

52. (small* and (real or detectable) and (change or difference)).ti,ab.

53. (meaningful change or "ceiling effect" or "floor effect" or "Item response model" or IRT or Rasch or "Differential item functioning" or DIF or "computer adaptive testing" or "item bank" or "cross-cultural equivalence").ti,ab.

54. error analysis/

55. exp test construction/

56. exp interrater reliability/

57. exp content analysis/

58. exp "error of measurement"/

59. exp factor structure/

60. exp testing methods/

61. exp statistical reliability/

62. prediction errors/ or "consistency (measurement)"/

63. or/25-62

64. 8 and 24 and 63

CINAHL EBSCO search strategy

Lines S10 to S41 (S42) is a translation of a validated search filter for finding studies on measurement properties for PubMed: Terwee CB, Jansma EP, Riphagen II, de Vet HCW. Development of a methodological PubMed search filter for finding studies on measurement properties of measurement instruments. Quality of Life Research 2009;18:1115-1123.

S1 (MH "Cerebrovascular Disorders") OR (MH "Basal Ganglia Cerebrovascular Disease+") OR (MH "Carotid Artery Diseases") OR (MH "Carotid Artery Dissections") OR (MH "Carotid Artery Thrombosis") OR (MH "Carotid Stenosis") OR (MH "Cerebral Ischemia") OR (MH "Cerebral Ischemia, Transient") OR (MH "Hypoxia-Ischemia, Brain") OR (MH "Cerebral Small Vessel Diseases") OR (MH "Cerebral Vasospasm") OR (MH "Cerebral Arterial Diseases") OR (MH "Cerebral Aneurysm") OR (MH "Intracranial Arteriosclerosis") OR (MH "Moyamoya Disease") OR (MH "Intracranial Embolism and Thrombosis+") OR (MH "Intracranial Hemorrhage") OR (MH "Subarachnoid Hemorrhage") OR (MH "Stroke+") OR (MH "Vertebral Artery Dissections")

S2 TI ((stroke or poststroke or post-stroke or cerebrovasc* or (cerebr* N3 vasc*) or CVA* or apoplectic or apoplex* or (transient N3 isch?emic N3 attack) or tia* or SAH or AVM or (cerebral small vessel N3 disease))) OR AB ((stroke or poststroke or post-stroke or cerebrovasc* or (cerebr* N3 vasc*) or CVA* or apoplectic or apoplex* or (transient N3 isch?emic N3 attack) or tia* or SAH or AVM or (cerebral small vessel N3 disease)))

S3 TI (((cerebr* or cerebell* or arteriovenous or vertebrobasil* or interhemispheric or hemispher* or intracran* or intracerebral or infratentorial or supratentorial or MCA* or ((anterior or posterior) N3 circulat*) or lenticulostriate or ((basilar or brachial or vertebr*) N3 arter*)) N3 (disease or damage* or disorder* or disturbance or dissection or lesion or syndrome or arrest or accident or lesion or vasculopathy or insult or attack or injury or insufficiency or malformation or obstruct* or anomal*))) OR AB (((cerebr* or cerebell* or arteriovenous or vertebrobasil* or interhemispheric or hemispher* or intracran* or intracerebral or infratentorial or supratentorial or MCA* or ((anterior or posterior) N3 circulat*) or lenticulostriate or ((basilar or brachial or vertebr*) N3 arter*)) N3 (disease or damage* or disorder* or disturbance or dissection or lesion or syndrome or arrest or accident or lesion or vasculopathy or insult or attack or injury or insufficiency or malformation or obstruct* or anomal*)))

S4 TI (((cerebr* or cerebell* or vertebrobasil* or interhemispheric or hemispher* or intracran* or corpus callosum or intracerebral or intracortical or intraventricular or periventricular or posterior fossa or infratentorial or supratentorial or MCA* or ((anterior or posterior) N3 circulation) or basal ganglia or ((basilar or brachial or vertebr*) N3 arter*) or space-occupying or brain ventricle* or subarachnoid* or arachnoid*) N3 (h?emorrhage or h?ematoma or bleed* or microh?emorrhage or microbleed or (encephalorrhagia or hematencephal*)))) OR AB (((cerebr* or cerebell* or vertebrobasil* or interhemispheric or hemispher* or intracran* or corpus callosum or intracerebral or intracortical or intraventricular or periventricular or posterior fossa or infratentorial or supratentorial or MCA* or ((anterior or posterior) N3 circulation) or basal ganglia or ((basilar or brachial or vertebr*) N3 arter*) or space-occupying or brain ventricle* or subarachnoid* or arachnoid*) N3 (h?emorrhage or h?ematoma or bleed* or microh?emorrhage or microbleed or (encephalorrhagia or hematencephal*))))

S5 TI (((cerebr* or cerebell* or arteriovenous or vertebrobasil* or interhemispheric or hemispher* or intracran* or corpus callosum or intracerebral or intracortical or intraventricular or periventricular or posterior fossa or infratentorial or supratentorial or MCA* or ((anterior or posterior) N3 circulation) or basal ganglia or ((basilar or brachial or vertebr*) N3 arter*) or space-occupying or brain ventricle* or lacunar or cortical or ocular) N3 (isch?emi* or infarct* or thrombo* or emboli* or occlus* or hypoxi* or vasospasm or obstruct* or vasculopathy or vasoconstrict*))) OR AB (((cerebr* or cerebell* or arteriovenous or vertebrobasil* or interhemispheric or hemispher* or intracran* or corpus callosum or intracerebral or intracortical or intraventricular or periventricular or posterior fossa or infratentorial or supratentorial or MCA* or ((anterior or posterior) N3 circulation) or basal ganglia or ((basilar or brachial or vertebr*) N3 arter*) or space-occupying or brain ventricle* or lacunar or cortical or ocular) N3 (isch?emi* or infarct* or thrombo* or emboli* or occlus* or hypoxi* or vasospasm or obstruct* or vasculopathy or vasoconstrict*)))

S6 TI (((carotid or cerebr* or cerebell* or intracranial or basilar or brachial or vertebr*) N3 (aneurysm or malformation* or dysplasia or disease or bruit or injur* or obstruct* or occlusion or constriction or presclerosis or scleros* or stenos* or atherosclero* or arteriosclero* or plaque* or thrombo* or embol* or arteriopathy))) OR AB (((carotid or cerebr* or cerebell* or intracranial or basilar or brachial or vertebr*) N3 (aneurysm or malformation* or dysplasia or disease or bruit or injur* or obstruct* or occlusion or constriction or presclerosis or scleros* or stenos* or atherosclero* or arteriosclero* or plaque* or thrombo* or embol* or arteriopathy)))

S7 (MH "Hemiplegia") OR (MH "Gait Disorders, Neurologic+")

S8 TI (hemipleg* or hemipar* or paresis or paraparesis or paretic) OR AB (hemipleg* or hemipar* or paresis or paraparesis or paretic)

S9 S1 OR S2 OR S3 OR S4 OR S5 OR S6 OR S7 OR S8

S10 (MH "Dysarthria") OR (MH "Articulation Disorders")

S11 (MH "Speech Articulation Tests")

S12 (MH "Speech Disorders")

S13 (MH "Voice Disorders")

S14 (MH "Aphonia")

S15 (MH "Dysphonia, Spasmodic")

S16 (MH "Dysphonia, Muscle Tension")

S17 (MH "Communicative Disorders")

S18 TI ((dysarth* OR dysphon* OR anarth* OR dyspros* OR aphon* OR dysfluen* OR stutter* OR stammer*)) OR AB ((dysarth* OR dysphon* OR anarth* OR dyspros* OR aphon* OR dysfluen* OR stutter* OR stammer*))

S19 TI (((speech OR articul* OR disarticul* OR phonat* OR phonolog* OR voice OR vocal OR prosod* OR intonat* OR respirat* OR communicat* OR fluen*) N5 (disorder* OR impair* OR problem* OR difficult*))) OR AB (((speech OR articul* OR disarticul* OR phonat* OR phonolog* OR voice OR vocal OR prosod* OR intonat* OR respirat* OR communicat* OR fluen*) N5 (disorder* OR impair* OR problem* OR difficult*)))

S20	TI ((speech N5 (slow* OR weak* OR imprecis* OR intelligibil* OR unintelligibil* OR accuracy OR fatigue))) OR AB ((speech N5 (slow* OR weak* OR imprecis* OR intelligibil* OR unintelligibil* OR accuracy OR fatigue)))

S21	(MH "Mouth+")

S22	(MH "Larynx+")

S23	(MH "Laryngeal Muscles")

S24	(MH "Pharynx")

S25	(MH "Pharyngeal Muscles")

S26	(MH "Palatal Muscles")

S27	TI ((mouth OR tongue OR lingual OR palat* OR laryn* OR pharyn* OR orofacial OR oro-facial OR “face musc*” OR “facial musc*”)) OR AB ((mouth OR tongue OR lingual OR palat* OR laryn* OR pharyn* OR orofacial OR oro-facial OR “face musc*” OR “facial musc*”))

S28	S21 OR S22 OR S23 OR S24 OR S25 OR S26 OR S27

S29	(MH "Movement Disorders")

S30	(MH "Ataxia")

S31	(MH "Dystonia")

S32	(MH "Dystonic Disorders")

S33	(MH "Hyperkinesis")

S34	(MH "Hypokinesia")

S35	(MH "Muscle Hypertonia+")

S36	(MH "Muscle Weakness")

S37	(MH "Muscular Diseases")

S38	(MH "Muscle Spasticity")

S39	TI ((atax* OR dyston* OR hyperkin* OR hypokin* OR hypoton* OR hyperton* OR flaccid* OR spastic*)) OR AB ((atax* OR dyston* OR hyperkin* OR hypokin* OR hypoton* OR hyperton* OR flaccid* OR spastic*))

S40	S29 OR S30 OR S31 OR S32 OR S33 OR S34 OR S35 OR S36 OR S37 OR S38 OR S39

S41	S28 AND S40

S42	S10 OR S11 OR S12 OR S13 OR S14 OR S15 OR S16 OR S17 OR S18 OR S19 OR S20 OR S41

S43	MH “Psychometrics”) or (TI psychometr* or AB psychometr*) or (TI clinimetr* or AB clinimetr*) or (TI clinometr* OR AB clinometr*) or (MH “Outcome Assessment”) or (TI outcome assessment or AB outcome assessment) or (TI outcome measure* or AB outcome measure*) or (MH “Health Status Indicators”) or (MH “Reproducibility of Results”) or (MH “Discriminant Analysis”) or ((TI reproducib* or AB reproducib*) or (TI reliab* or AB reliab*) or (

TI unreliab* or AB unreliab*)) or ((TI valid* or AB valid*) or (TI coefficient or AB coefficient) or (TI homogeneity or AB homogeneity)) or (TI homogeneous or AB homogeneous) or (TI “coefficient of variation” or AB “coefficient of variation”) or (TI “internal consistency” or AB “internal consistency”) or (MH “Internal Consistency+”) or (MH “Reliability+”) or (MH “Measurement Error+”) or (MH “Content Validity+”) or “hypothesis testing” or “structural validity” or “cross-cultural validity” or (MH “Criterion-Related Validity+”) or “responsiveness” or “interpretability” or (TI reliab* or AB reliab*) and ((TI test or AB test) OR (TI retest or AB retest)) or (TI stability or AB stability) or (TI interrater or AB interrater) or (TI inter-rater or AB inter-rater) or (TI intrarater or AB intrarater) or (TI intra-rater or AB intrarater) or (TI intertester or AB intertester) or (TI inter-tester or AB inter-tester) or (TI intratester or AB intratester) or (TI intra-tester or AB intra-tester) or (TI interobserver or AB interobserver) or (TI inter-observer or AB inter-observer) or (TI intraobserver or AB intraobserver) or (TI intra-observer or AB intra-observer) or (TI intertechnician or AB intertechnician) or (TI inter-technician or AB inter-technician) or (TI intratechnician or AB intratechnician) or (TI intra-technician or AB intra-technician) or (TI interexaminer or AB interexaminer) or (TI inter-examiner or AB inter-examiner) or (TI intraexaminer or AB intraexaminer) OR (TI intra-examiner or AB intra-examiner) or (TI intra-examiner or AB intraexaminer) or (TI interassay or AB interassay) or (TI inter-assay or AB inter-assay) or (TI intraassay or AB intraassay) or (TI intra-assay or AB intra-assay) or (TI interindividual or AB interindividual) or (TI inter-individual or AB inter-individual) OR (TI intraindividual or AB intraindividual) or (TI intra-individual or AB intra-individual) or (TI interparticipant or AB interparticipant) or (TI inter-participant or AB inter-participant) or (TI intraparticipant or AB intraparticipant) or (TI intra-participant or AB intra-participant) or (TI kappa or AB kappa) or (TI kappa’s or AB kappa’s) or (TI kappas or AB kappas) or (TI repeatab* or AB repeatab*) or (TI responsive* or AB responsive*) or (TI interpretab* or AB interpretab*)

S44 S9 AND S42 AND S43

APA PsycINFO Ovid search strategy

1. cerebrovascular disorders/ or cerebral hemorrhage/ or exp cerebral ischemia/ or cerebral small vessel disease/ or cerebrovascular accidents/ or subarachnoid hemorrhage/

2. (stroke or poststroke or post-stroke or cerebrovasc$ or (cerebr$ adj3 vasc$) or CVA$ or apoplectic or apoplex$ or (transient adj3 isch?emic adj3 attack) or tia$ or SAH or AVM or ESUS or ICH or (cerebral small vessel adj3 disease$)).tw.

3. ((cerebr$ or cerebell$ or arteriovenous or vertebrobasil$ or interhemispheric or hemispher$ or intracran$ or intracerebral or infratentorial or supratentorial or MCA$ or ((anterior or posterior) adj3 circulat$) or lenticulostriate or ((basilar or brachial or vertebr$) adj3 arter$)) adj3 ((blood adj5 clot$) or disease$ or damage$ or disorder$ or disturbance or dissection or lesion or syndrome or arrest or accident or lesion or vasculopathy or insult or attack or injury or insufficiency or malformation or obstruct$ or anomal$)).tw.

4. ((cerebr$ or cerebell$ or arteriovenous or vertebrobasil$ or interhemispheric or hemispher$ or intracran$ or corpus callosum or intracerebral or intracortical or intraventricular or periventricular or posterior fossa or infratentorial or supratentorial or MCA$ or ((anterior or posterior) adj3 circulation) or basal ganglia or ((basilar or brachial or vertebr$) adj3 arter$) or space-occupying or brain ventricle$ or lacunar or cortical or ocular) adj3 (isch?emi$ or infarct$ or thrombo$ or emboli$ or occlus$ or hypoxi$ or vasospasm or obstruct$ or vasoconstrict$)).tw.

5. ((cerebr$ or cerebell$ or vertebrobasil$ or interhemispheric or hemispher$ or intracran$ or corpus callosum or intracerebral or intracortical or intraventricular or periventricular or posterior fossa or infratentorial or supratentorial or MCA$ or ((anterior or posterior) adj3 circulation) or basal ganglia or ((basilar or brachial or vertebr$) adj3 arter$) or space-occupying or brain ventricle$ or subarachnoid$ or arachnoid$) adj3 (h?emorrhag$ or h?ematom$ or bleed$)).tw.

6. hemiparesis/ or hemiplegia/

7. (hemipleg$ or hemipar$ or paresis or paretic).tw.

8. or/1-7

9. dysarthria/ or articulation disorders/

10. dysphonia/ or speech disorders/

11. (dysarth$ or dyphon$ or anarth$ or dyspros$ or aphon$ or dysfluen$ or stutter$ or stammer$).tw.

12. ((speech or articul$ or disarticul$ or phonat$ or phonolog$ or voice or vocal or prosod$ or intonat$ or respirat$ or communicat$ or fluen$) adj5 (disorder$ or impair$ or problem$ or difficult$)).tw.

13. (speech adj5 (slow$ or weak$ or imprecis$ or intelligibil$ or unintelligibil$ or accuracy or fatigue)).tw.

14. "mouth (anatomy)"/ or tongue/

15. respiratory system/ or exp larynx/ or pharynx/

16. vocal cords/

17. facial muscles/

18. (mouth or tongue or lingual or palat$ or laryn$ or pharyn$ or orofacial or oro-facial or face musc$ or facial musc$).tw.

19. 14 or 15 or 16 or 17 or 18

20. muscular disorders/ or exp movement disorders/ or ataxia/ or bradykinesia/ or dyskinesia/ or hyperkinesis/ or neuromuscular disorders/ or spasms/ or muscle spasms/

21. (atax* or dyston* or hyperkin* or hypokin* or hypoton* or hyperton* or flaccid* or spastic*).tw.

22. 20 or 21

23. 19 and 22

24. 9 or 10 or 11 or 12 or 13 or 23

25. exp psychometrics/

26. statistics/

27. methodology/ or causal analysis/ or cohort analysis/ or content analysis/ or data collection/ or empirical methods/

28. exp experimental design/

29. psychometr*.ti,ab.

30. (clinimetr* or clinometr*).tw.

31. outcome assessment.ti,ab.

32. outcome measure*.tw.

33. observer variation.ti,ab.

34. reproducib*.ti,ab.

35. (reliab* or unreliab* or valid* or coefficient or homogeneity or homogeneous or "internal consistency").ti,ab.

36. (cronbach* and (alpha or alphas)).ti,ab.

37. (item and (correlation* or selection* or reduction*)).ti,ab.

38. (agreement or precision or imprecision or "precise values" or test-retest).ti,ab.

39. (test and retest).ti,ab.

40. (reliab* and (test or retest)).ti,ab.

41. (stability or interrater or inter-rater or intrarater or intra-rater or intertester or inter-tester or intratester or intra-tester or interobserver or inter-observer or intraobserver or intraobserver or intertechnician or inter-technician or intratechnician or intra-technician or interexaminer or inter-examiner or intraexaminer or intra-examiner or interassay or interassay or intraassay or intra-assay or interindividual or inter-individual or intraindividual or intra-individual or

interparticipant or inter-participant or intraparticipant or intra-participant or kappa or kappa's or kappas or repeatab*).ti,ab.

42. ((replicab* or repeated) and (measure or measures or findings or result or results or test or tests)).ti,ab.

43. (generaliza* or generalisa* or concordance).ti,ab.

44. (intraclass and correlation*).ti,ab.

45. (discriminative or "known group" or factor analysis or factor analyses or dimension* or subscale*).ti,ab.

46. (multitrait and scaling and (analysis or analyses)).ti,ab.

47. (item discriminant or interscale correlation* or error or errors or "individual variability").ti,ab.

48. (variability and (analysis or values)).ti,ab.

49. (uncertainty and (measurement or measuring)).ti,ab.

50. ("standard error of measurement" or sensitiv* or responsive*).ti,ab.

51. ((minimal or minimally or clinical or clinically) and (important or significant or detectable) and (change or difference)).ti,ab.

52. (small* and (real or detectable) and (change or difference)).ti,ab.

53. (meaningful change or "ceiling effect" or "floor effect" or "Item response model" or IRT or Rasch or "Differential item functioning" or DIF or "computer adaptive testing" or "item bank" or "cross-cultural equivalence").ti,ab.

54. error analysis/

55. exp test construction/

56. exp interrater reliability/

57. exp content analysis/

58. exp "error of measurement"/

59. exp factor structure/

60. exp testing methods/

61. exp statistical reliability/

62. prediction errors/ or "consistency (measurement)"/

63. or/25-62

64. 8 and 24 and 63

1. Mead, G.E., et al., *A systematic review and synthesis of global stroke guidelines on behalf of the World Stroke Organization*. International Journal of Stroke, 2023. **18**(5): p. 499-531.
2. Cheyne, J.D., *'Save the time of the user': developing a systematic search strategy for retrieving literature in stroke healthcare*, in *International Journal of Stroke*. 2023, SAGE Publications Inc.: Netherlands. p. 6.
3. Terwee, C.B., et al., *Development of a methodological PubMed search filter for finding studies on measurement properties of measurement instruments.* Qual Life Res, 2009. **18**(8): p. 1115-23.
4. Haddaway, N.R., M.J. Grainger, and C.T. Gray, *Citationchaser: A tool for transparent and efficient forward and backward citation chasing in systematic searching.* Research Synthesis Methods, 2022. **13**(4): p. 533-545.
