# Supplementary material for: How do we measure dysarthria after stroke? A systematic review to guide the core outcome set for dysarthria
Source: BMJ Open. 2025 May 23;15(5):e099662. doi: 10.1136/bmjopen-2025-099662 (PMC12104885; doi:10.1136/bmjopen-2025-099662)
Supplement: online supplemental file 4 [file bmjopen-15-5-s004.pdf]

Supplementary file 4

Table: Reason for exclusion following full text review of published information relating to measurement instrument

Name of measurement instrument	Related published references	Reason for exclusion
Acoustic and physiological voice assessment<br>Multi-dimensional voice program	(56-58)	Not available commercially
Bogenhausen Dysarthria Scales (BoDyS)	(59, 60)	German language assessment
E-learning based intelligibility test	(61)	Not available commercially<br>Unclear what language
Communication Effectiveness Survey (CES)	(62-64)	Only tested on neurodegenerative conditions not tested on stroke
Communication Profile for individuals with dysarthria	(65)	Unpublished & unable to locate any data
Communication Effectiveness Index for aphasia (CETI)	(66)	Designed & tested on aphasia only
Inpatient Functional Communication Interview (IFCI)	(37, 67)	Not a measurement for post-stroke dysarthria
National Institutes of Health Stroke Scale (NIHSS) (ref)	(45, 49, 68, 69)	Dysarthria included as part of a full screening for diagnosing unable to extract dysarthria specific data
Speech intelligibility Test<br>Diagnostic computer-based version of certain parts of the AIDS	(70)	No testing data<br>Referred back to the Assessment of intelligibility of dysarthric Speech AIDS data
Robertson Dysarthria Profile	(71)	No longer in print not available commercially
Speech intelligibility Test<br>Diagnostic computer-based version of certain parts of the AIDS	(70)	No testing data<br>Referred back to the Assessment of intelligibility of dysarthric Speech AIDS data
