# Supplementary material for: How do we measure dysarthria after stroke? A systematic review to guide the core outcome set for dysarthria
Source: BMJ Open. 2025 May 23;15(5):e099662. doi: 10.1136/bmjopen-2025-099662 (PMC12104885; doi:10.1136/bmjopen-2025-099662)
Supplement: online supplemental file 5 [file bmjopen-15-5-s005.pdf]

Supplementary file 5: Table: Overview of clinical utility and reliability/validity data of included measurement instruments for post-stroke dysarthria

**Name of screening measurement instrument & related papers**	**Total participants & diagnosis**	**Feasible to use clinically[a]**	**Validity & Reliability data[b]**	**Quality of evidence[c]**
Maximum phonation time (MPT) and maximum repetition rate (MRR)<br>(25)	n=130 participants<br>Stroke=26	• No specific training<br>• One clinician needed to understand and interpret the findings<br>• Aphasia accessible<br>• Few minutes<br>• Aphasia accessible<br>• No purchase required	Reported as an unreliable measure from the data available.<br>MPR – low sensitivity of 0.58 and specificity of 0.92<br>MPT sensitivity values (proportion of true positives) were 0.05 showing useless as a diagnostic marker of speech impairment<br>True negatives (specificity) were 1.0 for MPT	Very low due to small numbers of participants after stroke <30
**Name of diagnostic measurement instrument**	**Total participants & diagnosis**	**Feasible to use clinically[a]**	**Validity & Reliability data[b]**	**Quality of evidence[c]**
Assessment of intelligibility of dysarthric Speech AIDS<br>(40)	n=9<br>Mix of CVA and TBI combined	• No specific training required.<br>• Clinician would be required to carry out due to transcription.<br>• 2 people needed - examiner and judge who rates recorded samples<br>• Time taken not specified, includes 220 words to be transcribed, 50 word sample, 22 sentences<br>• Not aphasia accessible<br>• Easy to locate online for commercial purchase £ 154.80+ for assessment - add on cost of microphone and recorder	Reliability of single words Intra-judge Person product moment correlations (r) were .90 (multiple choice) and .87 (transcription)<br>Interjudge – no difference with multiple choice format (F=1.50, df 4, 32)<br>Significant difference transcription format (F=4.2, df 4, 32; p>0.01)<br>Sentences:<br>Interjudge – no significant difference between 4 judges intelligibile speech and rate of intelligible speech (F=.39 and 2.69 respectively df 3, 30)<br>Coefficients range from .93 to .99 for intelligibility and .99 for rate of intelligible speech.<br>Intrajudge correlations range from .96 to .99 for intelligibility and .99 for rate of intelligible speech.	Very low <30<br>Due to small number of participants involved in psychometric testing

Frenchay Dysarthria Assessment 2nd Edition<br>(27, 72-75)<br>Pending unpublished data from authors.	n=26 total<br>n=4 CVA	• No specific training to use test<br>• Single clinician needed with training as a speech and language therapist<br>• Able to interpret results<br>• No time given for time to carry out<br>• Some parts aphasia accessible but some reading required or words and sentences<br>• Easy to source for purchase<br>• Cost £199.14	No numerical results reported for stroke specific data in manual<br>Psychometric data available for other non-stroke conditions<br>Interjudge reliability as reported in the FDA (Enderby, 1983) test manual ranged from .79 to .92. Spearman's r correlation revealed moderate to high intrajudge reliability (rs = .85, .87, .90).<br>Interjudge reliability for the FDA as determined by Spearman's r correlation revealed moderate reliability among the three judges, with rs = .72, .72, and .77, p G .01.<br>FDA reliability of scores within listener reliability score 0.88 and between listener 0.68	Very low due to small numbers of participants after stroke <30
Iowa Oral Performance Instrument<br>(76)	n=18 total<br>n=3 CVA	• No specific training<br>• One clinician needed to understand and interpret the findings<br>• Aphasia accessible<br>• No time given as this will vary according to use<br>• Sourced quickly online to purchase<br>• No definitive prices shown, price on application online estimation $1200-$2000 & single use tongue bulbs	No data on validity of reliability of this instrument as a measure of dysarthria.<br><br>Test-re-test reliability of objective measure only ($F(1,12) = 6.83$, $p = .023$]	Unknown as no relevant data
Reading passages out loud to judge motor speech<br>(29, 77, 78)	n=15 total<br>n=9 CVA	• No specific training<br>• One clinician needed to understand and interpret the findings<br>• Not aphasia accessible reading required<br>• Time taken to read passage and interpret but no time specified<br>• No purchase required	No data on validity of reliability of this instrument as a measure of dysarthria.<br><br>Analysis of the passage itself rather than the validity & reliability of using this to assess dysarthria	Unknown as no relevant data
**Name of outcome measurement**	**Total participants & diagnosis**	**Feasible to use clinically[a]**	**Validity & Reliability data[b]**	**Quality of evidence[c]**

**instrument patient report**				
Communication Outcomes After STroke Scale (COAST) (30, 35)	n=102 CVA aphasia and/or dysarthria<br>n=30 dysarthria	• No specific training<br>• Could be introduced to patient by anyone<br>• Suggested completion time of 20-25 mins<br>• Aphasia accessible<br>• Quickly located online to obtain<br>• Free of charge on application	A revised scale of 20 items was produced, demonstrating good internal consistency and test–retest reliability (a=0.83–92; ICC=0.72–0.88).<br>Not designed to be repeated so no measure of responsiveness	Moderate due to participant numbers ≥100 patients (≥30 dysarthria)<br>Numerical data showing reliability & internal consistency
The Communicative Participation Item Bank (CPIB): item bank calibration and development of a disorder-generic short form. (79)	n=141<br>n= 18 CVA	• No specific training<br>• Could be introduced to patient by anyone<br>• Aphasia accessible with support<br>• No suggested completion time<br>• Easily accessible from publication	No data on validity of reliability of this instrument as a measure of post-stroke dysarthria.<br>The do report a significant effect of different diagnosis on communicative participation with large effect size: $F(3, 131) = 5.97$, $p = .001$, $r2 = .14$.	Very low due to small numbers of participants after stroke<30
Dysarthria Impact Profile (32)	n=31<br>n=7 CVA	• No specific training<br>• Could be introduced to patient by anyone<br>• Not Aphasia accessible requires reading<br>• No suggested completion time<br>• Easily accessible from publication	Internal consistency with values above 0.8 for Cronbach's α<br>Overall Intra-rater reliability strong for all sections of scale with pearson's correlation for all sections. Validity showed strong correlations between the sets of scores $r=0.683$, $p<0.01$	Very low due to small numbers of participants after stroke<30
Questionnaire on Acquired Speech Disorders (33)	n=55<br>n=1 CVA	• No specific training<br>• Could be introduced to patient by anyone<br>• Not Aphasia accessible requires reading<br>• No suggested completion time<br>• Easily accessible from publication	No data on validity of reliability of this instrument as a measure of dysarthria.<br>Data given on association with the Communication Profile scores for each participant. Reported relatively high association ($rs=0.683$, $p\leq0.01$). Correlations are generally moderate to high (0.4-0.7).	Very low due to small numbers of participants after stroke<30
Quality of Life for Dysarthric Speakers QOL-DyS (34)	n=50<br>n=7 CVA	• No specific training<br>• Could be introduced to patient by anyone<br>• Not Aphasia accessible requires reading	Overall Cronbach's coefficient reported as excellent $\alpha = 0.90$.<br>Intraclass correlation coefficient for the overall QOL-DyS score was 0.98 with 95% confidence interval from 0.97 to 0.99.	Very low due to small numbers of participants after stroke<30

		• No suggested completion time<br>• Easily accessible from publication		
**Name of outcome measurement instrument therapist report**	**Total participants & diagnosis**	• **Feasible to use clinically[a]**	**Validity & Reliability data[b]**	**Quality of evidence[c]**
OHW (O'Halloran, Hickson & Worrall) Scales for speech, language and cognitive communication rating scales (43)	n=38 total<br>n=11 dysarthria CVA	• Need to have carried out the IFCI<br>• May need to be familiar with IFCI, International Classification of Functioning and Health and OHW scales<br>• Experienced clinician<br>• No time for completion given<br>• Suitable for people with dysarthria and aphasia<br>• No purchase required, version in published article	Strong and significant concurrent criterion validity and significant interrater reliability<br>Interrater agreement was moderately high for the OHW speech and cognitive communicative scales but low for the OHW language scale. Interrater agreement on the OHW language scale requires further investigation.<br>Speech – absolute agreement on rating 70.8% weighted kappa .837<br>Speech concurrent validity with standardised measure (AIDS) .82	Very low due to small numbers of participants with dysarthria after stroke<30
Therapy Outcome Measures<br><br>(38, 80) Rating Conversations using the Therapy Outcome Measure (TOM) for aphasia/dysarthria	n=102 CVA aphasia and/or dysarthria<br>n=30 dysarthria	• Training not mandatory but recommended<br>• Clinician with expertise in that condition to judge<br>• Few minutes completion time<br>• Aphasia accessible<br>• Quickly located online to obtain the full manual £39.49	The manual indicates the Hesketh paper:<br>The intra-rater agreement was high: 93% of ratings were within a half point of each other on the TOM scale.<br>The intra class correlation (ICC) for intra-rater agreement was 0.92(Hesketh et al.,2008).-<br>Inter-rater agreement was slightly lower with 77% of ratings within a half point on the 11-point scale; ICC was 0.83(Hesketh et al.,2008).<br>Conversation reliability was equally good; 78% of the ratings were within a half point, with ICC being 0.82 (Hesketh et al., 2008: two videotaped interviews were conducted over a 2-week period.<br>All three comparisons (0.82-0.92) are well above commonly accepted levels for reliability data	Moderate due to participant numbers ≥100 patients (≥30 dysarthria)<br>Numerical data showing reliability & internal consistency

a- **Clinical utility: Training to use, training to interpret, people needed to carry out, completion time, accessible to people with aphasia, commercial availability and cost**
b- **Validity data and reliability data as reported**
c- **High** - Consistent findings in multiple studies of at least good quality OR one study of excellent quality AND a total sample size of ≥100 patients; **Moderate -** Conflicting findings in multiple studies of at least good quality OR consistent findings in multiple studies of at least fair quality OR one study of good quality AND a total sample size of ≥50 patients**; Low -** Conflicting findings in multiple studies of at least fair quality OR one study of fair quality AND a total sample size of ≥30 patients; **Very low** - Only studies of poor quality OR a total sample size of <30 patients; **Unknown** no studies (Prinsen ref 2016).
