# Supplementary material for: How do we measure dysarthria after stroke? A systematic review to guide the core outcome set for dysarthria
Source: BMJ Open. 2025 May 23;15(5):e099662. doi: 10.1136/bmjopen-2025-099662 (PMC12104885; doi:10.1136/bmjopen-2025-099662)
Supplement: Supplementary file 6 [file bmjopen-2025-099662.reviewer_comments.pdf]

PEER REVIEW HISTORY

BMJ Open publishes all reviews undertaken for accepted manuscripts. Reviewers are asked to complete a checklist review form and are provided with free text boxes to elaborate on their assessment. These free text comments are reproduced below.

ARTICLE DETAILS

Title (Provisional)

How do we measure dysarthria after stroke? A Systematic review to guide the Core Outcome Set for Dysarthria

Authors

Mitchell, Claire; El Kouaissi, Sabrina; Duncan-Zaleski, Martha; Bowen, Audrey; Conroy, Paul; Whelan, Brooke-Mai; Wallace, Sarah J. Jane; Cheyne, Joshua; Kirkham, Jamie J.

VERSION 1 - REVIEW

**Reviewer**	**1**
**Name**	**Luchesi Cera , Maysa**
**Affiliation**	**Universidade de Brasília**
**Date**	**22-Feb-2025**
**COI**	**None**

Thank you for giving me the chance to comment on this manuscript that identifies speech after stroke related measurement instruments, examines their clinical utility and understands their psychometric data.

The study found measurement instruments with uncertain quality of evidence and three have been suggested for inclusion with a Core Outcome Set.

It may be extremely helpful for the authors to include additional information in the manuscript.

usar o termo Core Outcome Set de modo mais padronizado e revisara apresentação da sigla.

Background:

The introduction does not make clear the scientific knowledge currently available on the topic, nor does it specify the lack. Therefore, it is important that the justification be better supported.

It would be important to present:

a. The definition of dysarthria,

b. Definition and clinical utility of Core Outcome Set. Would they be screening measures? The text does not make it clear.

c. More information in the explanation about the Core Outcome Set for dysarthria;

d. Reference whether there are reviews on screening for dysarthria, diagnostic assessment of dysarthria, as well as clarify what information is already available in the literature.

Additionally, use the term Core Outcome Set in a more standardized way and review the presentation of the acronym.

Methods:

1. Systematic reviews should include studies published in different languages. However, an exclusion criterion was non-English language studies or Searching instruments in other languages. In any language, dysarthria has the same definition and the same diagnostic criteria. The difference related to the languages would not be detrimental; on the contrary, it would make this review even more relevant.

2. Was the Grey literature searched?

I hope these comments are beneficial in enhancing the quality of the manuscript.

Reviewer 2
Name Kelly, Crystal
Affiliation Griffith University
Date 25-Feb-2025
COI None

This article provides a comprehensive and detailed systematic review of dysarthria measurement instruments for people following stroke which is a highly relevant and valuable contribution to the field. The clarity of your methodology and inclusion criteria is strong, and the use of COSMIN and GRADE to assess the quality of instruments is well-conceptualised. However, the manuscript could benefit from some organisational adjustments, such as providing clearer transitions between sections, condensing repetitive information, and enhancing readability by simplifying some of the longer sentences. Additionally, being more explicit about limitations and next steps in future research would strengthen the Discussion and conclusion sections. Overall, your work lays an important foundation for better understanding and assessing dysarthria after stroke.

Manuscript feedback

Location	Correction/suggestion
Pg. 4, line 7	Measurement instruments for dysarthria after stroke do currently exist, however ~~already exist but we don't know if~~ it is unknown whether any of these will relate to the core outcomes identified in COS-Speech.
Pg. 4, line 10-12	A systematic review will assist in identifying all possible instruments and consider the extent to which these meet the needs of COS-Speech.
Pg. 4, line 40	Expand on *why* these changes were made and how they might influence the results (e.g excluding instruments that assessed dysarthria as part of a larger battery). In addition, the article states it follows PRISMA standards, but readers may appreciate a sentence detailing how many reviewers performed screening, data extraction, and risk-of-bias assessment. You do mention the number of reviewers, but linking it explicitly to PRISMA items is helpful for transparency.
Pg. 4, Line 42	The research team carried out…
Pg. 4, Line 51	In addition, the research team hand-searched
Pg. 5, Line 9	Detailed search terms combined all possible ~~terms~~ words or phrases for: (construct) dysarthria and (population) adult stroke and (instrument) measurement. ~~and we used T~~The

	relevant filters for measurement properties following COSMIN guidance was also used (15).
Pg. 5, lines 18-20	~~This meant we excluded~~ Aphasia only studies and other types of brain injury causing dysarthria including progressive neurological conditions and Traumatic Brain Injury were excluded from the current study.
Pg. 5, Lines 25-27	Suggest rewording sentence e.g. All quantitative study types which examined instruments developed or used for dysarthria following stroke were included in the review. Some of these study types included; standardisation, validation, diagnostic, cohort, case-control, experimental and randomised control trial.
Pg. 5, lines 29-38	Very lengthy sentence, consider splitting into multiple sentences.
Pg. 5, Lines 38-40	This sentence requires rewording. Also, further explanation of why this decision was made and potential implications is needed.
Pg. 6, lines 3-5	Authors of all included measurement instruments were contacted to provide ~~-~~any unpublished data that was relevant to the reliability or validity of their instrument that ~~we~~ could be used in this review.
Pg. 6, Line 23	Consider rephrasing: The ratings were classified as follows:
Pg. 6, lines 47-50	You mention that stroke survivors were involved but did not interpret results or contribute to writing/editing. It would strengthen the paper to clarify how their perspectives influenced the study.
Pg. 6, Line 60	Consider rephrasing: The Cochrane search identified 8,286 studies after removing duplicates.
Pg. 7, line 29	In the list of outcome measures, note that the two clinician-reported instruments are cited as "(12, 37, 38)" even though that suggests three references for two instruments. Verify that the citations correctly correspond to each instrument.
Pg. 8, Line 40	IOPI ~~IPOI~~
Pg. 9, lines 26-29	The CIPB, QASD, QOL-DyS, DIP, and OHW were tested in stroke populations of n=18, n=1, n=7, n=7, and n=11, respectively.
Pg. 10, line 33	Further detail included in this section to summarise the results reported would strengthen this section.
Discussion section	Including additional information to the discussion would strengthen the article overall. E.g. outline the gaps in existing measurement instruments, the clinical and research implications of recommending FDA, COAST and D-TOMS, as well as future research directions (e.g. validating these tools in larger stroke populations).<br><br>Early in the Discussion, emphasize the *novelty* of your findings: for example, that this is the first systematic review to align dysarthria measures with the newly defined COS-Speech domains.

	You outline the measures recommended (FDA, COAST, D-TOMs) but could further elaborate on *how* clinicians might practically incorporate them or what challenges might arise.
Pg. 14, line 10	In the final paragraph, you mention the importance of developing or adapting measures for communication partners. Expanding on which existing instruments might be most easily adapted (or describing any preliminary work in that area) could strengthen your conclusion.

Reviewer **3**
Name **Yan, Nan**
Affiliation **University Town of Shenzhen**
Date **02-Mar-2025**
COI **None**

This study conducted a comprehensive investigation into measurement tools for four core outcome domains of post-stroke dysarthria: 1) intelligibility of speech, 2) ability to participate in conversations, 3) living well with dysarthria, 4) skills and knowledge of communication partners (where relevant). While the manuscript demonstrates methodological rigor through its application of COSMIN consensus standards and COMET guidelines for assessing measurement properties, with Frenchay Dysarthria Assessment II, Post-Stroke Communication Outcomes Scale, and Dysarthria Treatment Outcome Measure being identified as most relevant, several limitations warrant consideration:

Instrument Selection Rationale: The exclusion of established assessments like the ASHA-FACS Functional Communication Measure, which evaluates functional communication in real-life contexts, and the Voice-Related Quality of Life Survey remains unexplained. These instruments' omission requires justification given their validated application in adult communication assessment.

Clinical Translation Gap: Although the authors elaborate on scale selection criteria, the practical implementation framework for applying these measures to guide dysarthria rehabilitation remains underdeveloped. Specifically, the manuscript lacks actionable insights on how assessment outcomes could inform personalized therapeutic strategies or rehabilitation protocols.

Stroke-Specific Considerations: The rationale for focusing exclusively on post-stroke dysarthria requires strengthening. Given the condition's marked heterogeneity stemming from diverse neuroanatomical lesions, the selected generic functional scales may adequately capture impairment profiles but offer limited utility in directing lesion-specific rehabilitation approaches. This contrasts with stroke-induced dysarthria's unique pathophysiological complexity compared to other dysarthria subtypes.

Reviewer **4**
Name **Chiaramonte, Rita**

**Affiliation**	**University of Catania, Department of Physical Medicine and Rehabilitation**
**Date**	**02-Mar-2025**
**COI**	**None**

This study aims to systematically search for and identify all speech-related measurement instruments used after stroke.

Here my comments:

Methods: Add PICOS criteria.

Results: Include a table summarizing the authors, study design, sample characteristics, diagnostic measures, statistical outcomes, and conclusions of the included studies.

Discussion: Regarding the study's aim of assessing "clinical utility," please briefly explain all rehabilitation strategies currently used (DOI: 10.23736/S1973-9087.20.06242-5).

Conclusion: Clearly state the conclusion without introducing new references.

VERSION 1 - AUTHOR RESPONSE

Reviewer 1	
use the term Core Outcome Set in a more standardized way and review the presentation of the acronym	Thanks, we have now defined the COS term in a clearer way in the introduction and reviewed the presentation of the acronym hopefully making this easier to follow. Page 4.
To include in the introduction:<br>The definition of dysarthria	We have enhanced our definition of dysarthria in the background information. Page 4.
Definition and clinical utility of Core Outcome Set. Would they be screening measures? The text does not make it clear.	We have attempted to make clearer on page 4. Although a COS is a set of outcome measures rather that a set of screening measures it is possible that some measures used from screening might also be useful outcome measures. We have now added in wording to explain that they could be included in a COS, also page 4.
More information in the explanation about the Core Outcome Set for dysarthria	We have added in additional information to explain this more clearly. Page 4.
Reference whether there are reviews on screening for dysarthria, diagnostic assessment of dysarthria, as well as clarify what information is already available in the literature.	We have highlighted existing reviews of dysarthria measures and have highlighted relevant literature in the background. Page 4.
Methods:	
Systematic reviews should include studies published in different languages. However, an exclusion criterion was non-English language studies or Searching instruments in other	Thanks for this point which we do agree with in general for systematic reviews, especially reviews of effectiveness of interventions.

languages. In any language, dysarthria has the same definition and the same diagnostic criteria. The difference related to the languages would not be detrimental; on the contrary, it would make this review even more relevant.	However a Core Outcome Set needs to be generalisable and to highlight the core set. A COS does not preclude additional measures and measures specific to a language or culture can always be added to a COS by anyone running a trial with a target population where this would be relevant.<br>Well-established, robust measures for dysarthria can be relatively straight-forward to translate and adapt to other languages in response to the COS.<br>We appreciate this is a potential limitation of this review which is mentioned in the strengths and limitations and conclusions. Page 4 and 14.
Was the Grey literature searched?	We didn't think we could claim this was the case as although we were willing to consider grey literature we didn't actively seek it. We did email all author of included measures to seek any unpublished data that included psychometric data related to their measure.<br>This is explained in the searching section of the manuscript page 6.
Reviewer 2	
However, the manuscript could benefit from some organisational adjustments, such as providing clearer transitions between sections, condensing repetitive information, and enhancing readability by simplifying some of the longer sentences.	Thank you, we have now re-visited all of the longer sentences in an attempt to enhance readability.<br>We have attempted to make transitions clear and reduce repetition.
Additionally, being more explicit about limitations and next steps in future research would strengthen the Discussion and conclusion sections.	We have included more detail in the discussion section and hope this strengthens the relevant section offering more information regarding next steps.
Reviewer 3	
Instrument Selection Rationale: The exclusion of established assessments like the ASHA-FACS Functional Communication Measure, which evaluates functional communication in real-life contexts, and the Voice-Related Quality of Life Survey remains unexplained. These instruments' omission requires justification given their validated application in adult communication assessment.	Thanks for raising, but interestingly in relation to ASHA-FACS, as far as our searching suggested this was not developed or used specifically for dysarthria following stroke as per our inclusion/exclusion criteria. There was no evidence of psychometric data for a dysarthria stroke population that we found, however we would be willing to amend this in light of any psychometric data we may have missed related to this study and its use in a post-stroke dysarthria population.<br>This is the same for the Voice-related QOL, which although it may be used with adults – we did not find reliability and validity data related to adults with post-stroke dysarthria in our searching so was excluded early on as developed for voice not post-stroke dysarthria.

	We believe we have included all eligible measures, but this review was quite specific and relates to evidence around development of measurement instruments in a specific dysarthric stroke population.
Clinical Translation Gap: Although the authors elaborate on scale selection criteria, the practical implementation framework for applying these measures to guide dysarthria rehabilitation remains underdeveloped.<br>Specifically, the manuscript lacks actionable insights on how assessment outcomes could inform personalized therapeutic strategies or rehabilitation protocols.	Agreed that we don't address how to apply these measures to guide dysarthria rehabilitation. This was never the intention of this paper as we set out to consider what relevant measurement instruments could be used with our Core Outcome Set.<br>Future research should absolutely consider how outcomes could inform rehabilitation.
The rationale for focusing exclusively on post-stroke dysarthria requires strengthening. Given the condition's marked heterogeneity stemming from diverse neuroanatomical lesions, the selected generic functional scales may adequately capture impairment profiles but offer limited utility in directing lesion-specific rehabilitation approaches. This contrasts with stroke-induced dysarthria's unique pathophysiological complexity compared to other dysarthria subtypes.	Agreed and in many ways your point supports our findings, that we could only find generic broad measurement instruments through our searching.<br>The included instruments were mainly devised for neurological dysarthria as a whole, certainly not offering much nuance on different presentations of dysarthria after stroke.<br>Our future directions of research absolutely suggest that more nuanced and in-depth measurement instruments are needed. We have tried to highlight where further research is needed in our discussion page 12-14.
Reviewer 4	
Methods: Add PICOS criteria.	PICO criteria now included. Population, intervention and study design is now included in the inclusion/exclusion criteria section. Page5.
Results: Include a table summarizing the authors, study design, sample characteristics, diagnostic measures, statistical outcomes, and conclusions of the included studies.	Thanks for this suggestion, we have included a table in supplementary file 5 (incorrectly labelled previously) that provides this information.
Discussion: Regarding the study's aim of assessing "clinical utility," please briefly explain all rehabilitation strategies currently used (DOI: 10.23736/S1973-9087.20.06242-5).	The purpose of this review was not related to the utility of rehabilitation strategies but the 'clinical utility' of the measures as explained in the OMERACT guidance.<br>This is explained in the searching section Page 7 and relates to practical and feasible issues related to how easy it is to carry out or do you need to attend training, is more than one person needed to carry it out, is it suitable for people with dysarthria and aphasia, is it very expensive – all practical clinical considerations. It would be beyond the scope of this COS paper to describe or review rehabilitation strategies, but the reader could be referred to the Cochrane review of interventions.

Conclusion: Clearly state the conclusion without introducing new references.	Now amended and the opening sentence of the discussion hopefully states this more clearly now without any new references. Page 11.

Manuscript Feedback Table:

**Location**	**Correction/suggestion**	
Pg. 4, line 7	Measurement instruments for dysarthria after stroke do currently exist, however ~~already~~ ~~exist~~ ~~but we don't know if~~ it is unknown whether any of these will relate to the core outcomes identified in COS-Speech.	Amended
Pg. 4, line 10-12	A systematic review will assist in identifying all possible instruments and consider the extent to which these meet the needs of COS-Speech.	Amended
Pg. 4, line 40	Expand on *why* these changes were made and how they might influence the results (e.g excluding instruments that assessed dysarthria as part of a larger battery). In addition, the article states it follows PRISMA standards, but readers may appreciate a sentence detailing how many reviewers performed screening, data extraction, and risk-of-bias assessment. You do mention the number of reviewers, but linking it explicitly to PRISMA items is helpful for transparency.	Expanded on why these changes were made and now amended in this inclusion/exclusion criteria section.<br><br>This information is now linked to PRISMA and the related item.
Pg. 4, Line 42	The research team carried out…	Amended
Pg. 4, Line 51	In addition, the research team hand-searched	Amended
Pg. 5, Line 9	Detailed search terms combined all possible ~~terms~~ words or phrases for: (construct) dysarthria and (population) adult stroke and (instrument) measurement. ~~and we used T~~The relevant filters for measurement properties following COSMIN guidance was also used (15).	Amended

Pg. 5, lines 18-20	This meant we excluded Aphasia only studies and other types of brain injury causing dysarthria including progressive neurological conditions and Traumatic Brain Injury were excluded from the current study.	Amended
Pg. 5, Lines 25-27	Suggest rewording sentence e.g. All quantitative study types which examined instruments developed or used for dysarthria following stroke were included in the review. Some of these study types included; standardisation, validation, diagnostic, cohort, case-control, experimental and randomised control trial.	Amended
Pg. 5, lines 29-38	Very lengthy sentence, consider splitting into multiple sentences.	Agreed, now split into multiple sentences and repetition removed.
Pg. 5, Lines 38-40	This sentence requires rewording. Also, further explanation of why this decision was made and potential implications is needed.	Re-worded, repetition removed and we have explained the potential implications.
Pg. 6, lines 3-5	Authors of all included measurement instruments were contacted to provide any unpublished data that was relevant to the reliability or validity of their instrument that we could be used in this review.	Amended
Pg. 6, Line 23	Consider rephrasing: The ratings were classified as follows:	Amended
Pg. 6, lines 47-50	You mention that stroke survivors were involved but did not interpret results or contribute to writing/editing. It would strengthen the paper to clarify how their perspectives influenced the study.	Thanks for the suggestion, now included.
Pg. 6, Line 60	Consider rephrasing: The Cochrane search identified 8,286 studies after removing duplicates.	Amended
Pg. 7, line 29	In the list of outcome measures, note that the two clinician-reported instruments are cited as	Checked and we can confirm that these are all correct.

	"(12, 37, 38)" even though that suggests three references for two instruments. Verify that the citations correctly correspond to each instrument.	Several of the measurement instruments have data in more than one study, all related references are mentioned and correspond to each instrument.
Pg. 8, Line 40	IOPI ~~IPOI~~	Amended
Pg. 9, lines 26-29	The CIPB, QASD, QOL-DyS, DIP, and OHW were tested in stroke populations of n=18, n=1, n=7, n=7, and n=11, respectively.	Amended
Pg. 10, line 33	Further detail included in this section to summarise the results reported would strengthen this section.	Further details now included in this section.
Discussion section	Including additional information to the discussion would strengthen the article overall. E.g. outline the gaps in existing measurement instruments, the clinical and research implications of recommending FDA, COAST and D-TOMS, as well as future research directions (e.g. validating these tools in larger stroke populations).<br><br>Early in the Discussion, emphasize the *novelty* of your findings: for example, that this is the first systematic review to align dysarthria measures with the newly defined COS-Speech domains.<br><br>You outline the measures recommended (FDA, COAST, D-TOMs) but could further elaborate on *how* clinicians might practically incorporate them or what challenges might arise.	Thanks for this helpful suggestion, the gaps are now included.<br><br>Now amended in opening sentence.<br><br>Further information now included to support clinicians in using the COS-Speech.
Pg. 14, line 10	In the final paragraph, you mention the importance of developing or adapting measures for communication partners. Expanding on which existing instruments might be most easily adapted (or describing	Thank you, now expanded and further suggestions made about adapting existing measures and where further development is needed.

	any preliminary work in that area) could strengthen your conclusion.	

VERSION 2 - REVIEW

Reviewer 1

Name Luchesi Cera , Maysa

Affiliation Universidade de Brasília

Date 16-Apr-2025

COI

The authors have responded to my comments.

The general clinical presentation of dysarthria may be fundamental for the understanding of readers unfamiliar with the area.
